# A Photoresponsive Stiff‐Stilbene Ligand Fuels the Reversible Unfolding of G‐Quadruplex DNA

**DOI:** 10.1002/anie.201900740

**Published:** 2019-02-20

**Authors:** Michael P. O'Hagan, Susanta Haldar, Marta Duchi, Thomas A. A. Oliver, Adrian J. Mulholland, Juan C. Morales, M. Carmen Galan

**Affiliations:** ^1^ School of Chemistry University of Bristol Cantock's Close BS8 1TS UK; ^2^ Instituto de Parasitología y Biomedicina “López Neyra” Consejo Superior de Investigaciones Científicas (CSIC) PTS Granada Avenida del Conocimiento 17 18016 Armilla Granada Spain

**Keywords:** circular dichroism, DNA, G-quadruplexes, oligonucleotides, photoregulation

## Abstract

The polymorphic nature of G‐quadruplex (G4) DNA structures points to a range of potential applications in nanodevices and an opportunity to control G4 in biological settings. Light is an attractive means for the regulation of oligonucleotide structure as it can be delivered with high spatiotemporal precision. However, surprisingly little attention has been devoted towards the development of ligands for G4 that allow photoregulation of G4 folding. We report a novel G4‐binding chemotype derived from stiff‐stilbene. Surprisingly however, whilst the ligand induces high stabilization in the potassium form of human telomeric DNA, it causes the unfolding of the same G4 sequence in sodium buffer. This effect can be reversed on demand by irradiation with 400 nm light through deactivation of the ligand by photo‐oxidation. By fuelling the system with the photolabile ligand, the conformation of G4 DNA was switched five times.

Reversible regulation of nucleic acid structure is a thriving area of research, and many DNA‐based switches have been reported over the past decade.^1^ G‐quadruplexes (G4) are a class of four‐stranded oligonucleotide secondary structures that form from sequences rich in guanine.^2^ These fascinating structures have garnered interest from across many scientific disciplines because of their structural polymorphism,^3^ diverse roles in biology,^4^ and applications as therapeutic targets,^5^ catalysts,^6^ and as the basis of functional nanodevices.^7^ Switchable control of G‐quadruplex topology offers exciting opportunities to further many of these applications, and a number of groups have demonstrated the regulation of DNA secondary structures by a variety of chemical triggers including pH^8^ and metal ions.^9^ Light offers significant advantages over chemical stimuli as it can be delivered with high spatiotemporal precision, allowing an additional level of control over the system.^10^ Previously, the groups of Ogasawara^11^ and Heckel^12^ have demonstrated the photoresponsive formation of G4 architectures through the incorporation of photoswitchable moieties within the oligonucleotide sequence. However, the requirement to engineer unnatural functionality into the biomolecule perhaps limits the scope of potential applications of these systems. Reversible regulation of G4 through a supramolecular approach, by employing a photoresponsive small‐molecule ligand as a fuel, would allow complementary applications to be realized, particularly in situations where pre‐modification of the nucleotide sequence is undesirable. The small number of light‐triggered G4 ligands developed to date are mainly engineered to cause irreversible covalent modification of the DNA structure upon photoirradiation.^13^ A notable exception is an azobenzene derivative developed by Wang and co‐workers that permits photoregulation of G4 folding and dissociation in aqueous media by isomerization of the azobenzene scaffold between the *cis* and the *trans* forms.^14^ However, the effects are significantly diminished under physiologically relevant ionic conditions where the conformational preference exerted by the high concentration of monovalent cations appears more difficult to overcome with a ligand‐driven approach.^15^

During the course of our studies on the development of novel G‐quadruplex ligands,^16^ we became interested in the potential of stiff‐stilbene to serve as a scaffold for a new class of photoresponsive G4‐binding molecules. Stiff‐stilbenes have recently been incorporated into the backbone of oligonucleotide hairpins to regulate DNA hybridization.^17^ However, to the best of our knowledge, use of this interesting scaffold as the basis of selective DNA‐targeting small molecules has not yet been explored.

Towards this end, (*E*)‐**1** and (*Z*)‐**1** were prepared as their diiodide salts in a three‐step procedure (Scheme 1). Following isolation, the ability of (*E*)‐**1** and (*Z*)‐**1** to induce thermal stability in G4 DNA was evaluated by means of a FRET melting assay (see the Supporting Information for details).

**Scheme 1:**
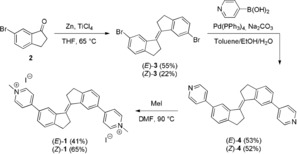
Synthesis of the pyridinium stiff‐stilbenes (*E*)‐**1** and (*Z*)‐**1**.

The results (Table 1) show that (*E*)‐**1** induces a high level of thermal stabilization (Δ*T*_m_=21.4 °C at 1 μm ligand) in the human telomeric G4 (F21T) in K^+^‐rich conditions, with the degree of stabilization increasing further as the concentration of (*E*)‐**1** is increased (Δ*T*_m_=26.3 °C at 2 μm). The thermal stability of F21T in Na^+^ conditions (Δ*T*_m_=11.8 °C) and of the FmycT (Δ*T*_m_=15.8 °C) sequence is also significantly increased by the addition of (*E*)‐**1**. Critically, the stability of duplex DNA is largely unaffected (Δ*T*_m_=0.8 °C at 1 μm), demonstrating the specificity of the ligand for the four‐stranded structure.

**Table 1:** Thermal stabilization (Δ*T*_m_) induced in G4 and duplex DNA by increasing concentrations of (*E*)‐**1**.^[a]^

	Δ*T*_m_ [°C]	
DNA species	1 μm (*E*)‐**1**	2 μm (*E*)‐**1**
F21T (K^+^)	21.4±0.3	26.3±0.7
F21T (Na^+^)	11.8±0.6	18.5±0.7
FmycT (K^+^)	15.8±0.8	23.2±1.1
F10T (K^+^)	0.8±0.2	2.1±0.2

[a] F21T=5′‐FAM‐GGGTTAGGGTTAGGGTTAGGG‐TAMRA‐3′; FmycT=5′‐FAM‐TTGAGGGTGGGTAGGGTGGGTAA‐TAMRA‐3′; F10T=5′‐FAM‐TATAGCTATA‐HEG‐TATAGCTATA‐TAMRA‐3′.

To confirm the ability of (*E*)‐**1** to discriminate between G4 and duplex DNA, we performed the assay against F21T (K^+^) under competitive conditions of increasing concentrations of unlabelled duplex DNA (Figure 1 and Table S1). At a 25‐fold molar excess of duplex DNA relative to G4 (corresponding to 325 duplex base pairs per G‐quadruplex), over 70 % of the induced thermal stabilization of F21T by (*E*)‐**1** is retained, demonstrating the high selectivity of (*E*)‐**1** for the G4 sequence. (*Z*)‐**1** also demonstrates significant and selective stabilization of G4 DNA (Table S2), although the induced thermal stabilizations are significantly lower than those of (*E*)‐**1** (e.g., Δ*T*_m_=9.7 °C for F21T in K^+^ conditions at 1 μm). These results demonstrate that stiff‐stilbene is a highly effective scaffold for the further development of potent and selective G4‐binding agents.

**Figure 1:**
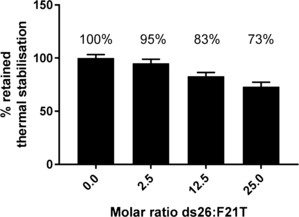
Retained thermal stabilization of F21T by 1 μm (*E*)‐**1** in the presence of increasing concentrations of ds26, a competitor duplex DNA hairpin.

To confirm the affinity of (*E*)‐**1** for G‐quadruplex DNA, we characterized the UV absorbance of the ligand (Figure S3) and performed a UV/Vis titration study against the unlabelled human telomeric sequence telo23 in K^+^ buffer (Figure S4). The resulting isotherms are not well‐described by simple 1:1 or 1:2 binding models, suggesting a more complex mode of interaction. However, the saturation of binding at low DNA concentration (approximately 3 μm) indicates affinity at the low micromolar level.

In order to examine the effects of (*E*)‐**1** on G4 topology and provide preliminary insight into the possible binding mode of (*E*)‐**1**, circular dichroism (CD) experiments were conducted using the human telomeric G4 sequence telo23, which forms a hybrid G4 topology in the presence of either K^+^ or Na^+^ ions (Figure 2 a).^18^ As expected, no CD was observed for the free ligand in the absence of DNA (Figure S5). Although no conformational change was observed in K^+^ conditions (Figure 2 b), binding was evidenced by a hyperchromic shift in the positive band at 288 nm. A plot of the change in ellipticity of this band (Figure S6) reveals that the inflection point occurs at 3 equivalents of (*E*)‐**1,** indicating a 3:1 binding stoichiometry. Furthermore, positive induced circular dichroism (ICD) signals are visible in the ligand region, which have previously been reported to indicate a groove binding mode of interaction rather than end stacking.^19^ The *E*‐(**1**)/telo23 system behaves remarkably differently in Na^+^ buffer, where increasing quantities of (*E*)‐**1** result in dramatic changes in the CD signature of the oligonucleotide, indicative of a conformational change (Figure 2 c). In the absence of ligand, the spectrum is characterized by positive bands at 295 nm and 245 nm and a negative band at 265 nm. Adding increasing amounts of (*E*)‐**1** causes a marked decrease in intensity of all three G4 bands, accompanied by the induction of a strong positive band at 273 nm. Dramatic induced dichroism in the ligand region (*λ*_max_=340 nm) is also observed, indicative of induced chirality in the ligand upon binding to DNA. These data show that (*E*)‐**1** remodels the hybrid G4 formed in Na^+^ buffer to an alternative structure. The changes take place on the timescale (minutes) of the CD experiment, suggesting an intramolecular rearrangement. Indeed, a kinetic plot of the evolution of the positive feature at 273 nm immediately following the addition of 10 equivalents of (*E*)‐**1** reveals a characteristic unfolding time of 245 s upon fitting to a single exponential function (Figure S7), comparable with ligand‐induced topological changes in DNA secondary structure reported by others.^20^ Ligand (*E*)‐**1** exerts a similar effect on the related sequence telo22 (Figure S8), which forms instead an antiparallel basket‐type G4 in Na^+^ conditions despite only differing from telo23 by the omission of the terminal thymine residue.^18^

**Figure 2:**
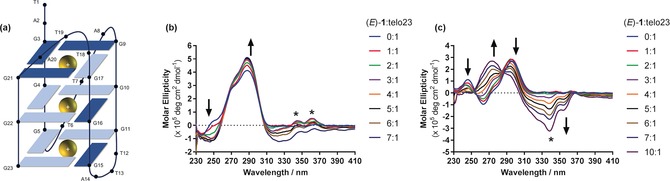
a) Schematic representation of the hybrid telo23 (5′‐TAGGGTTAGGGTTAGGGTTAGGG‐3′) G4 formed in K^+^ and Na^+^ conditions. b, c) Circular dichroism titrations of (*E*)‐**1** into telo23 in 100 mm b) K^+^ phosphate and c) Na^+^ phosphate buffer. Induced circular dichroism signals in the ligand are marked with *.

To gain structural insight into the nature of the conformational remodelling induced by (*E*)‐**1**, we performed ^1^H NMR experiments (Figure 3 a). Upon adding 2 equivalents of (*E*)‐**1**, a marked decrease in intensity of the imino signals associated to the guanine moieties at *δ*=10.4–11.8 ppm is observed, resulting in the disappearance of several resonances. No additional imino signals were observed to indicate folding to alternative G4 structures. In the context of the CD results, which show attenuation of all three bands corresponding to G4 secondary structure, this suggests that (*E*)‐**1** induces the unfolding of the G4 structure adopted by telo23 in Na^+^ conditions. Broadening of the non‐exchangeable aromatic resonances is also observed (Figure 3 a and Figure S9), indicating the conformational heterogeneity of the unfolded DNA. Diffusion‐ordered NMR experiments demonstrate that the diffusion coefficient of the oligonucleotide is unaffected by (*E*)‐**1** (Figure S10), indicating that the observed effects are not a result of ligand‐induced aggregation. The NMR and CD observations are further supported by well‐tempered metadynamics simulations, which display groove‐binding and intercalative interaction modes and show that (*E*)‐**1** induces the unfolding of the telomeric quadruplex oligonucleotide to a single‐stranded helix after 800 ns (Figure 3 b and the Supporting Information).^21^ To the best of our knowledge, only very rare examples of G4 disruption by small molecules have been reported previously.^22^

**Figure 3:**
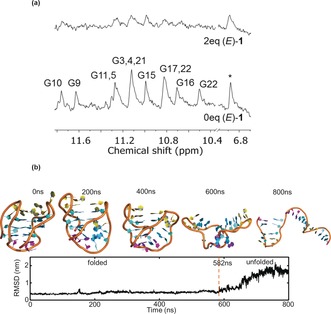
a) Partial ^1^H NMR spectra of telo23 in Na^+^ buffer in the absence (bottom) and presence (top) of 2 equivalents (*E*)‐**1**. Guanine imino proton assignments correspond to the labelling in Figure 2 a.^18^ A representative non‐exchangeable aromatic signal is marked with *. b) Well‐tempered metadynamics simulations showing unfolding of telomeric G4 by (*E*)‐**1** (for clarity, (*E*)‐**1** is not shown).

Having demonstrated the ability of (*E*)‐**1** to induce a rapid conformational rearrangement in telo23 G4, our attention turned towards exploring the photochemistry of the ligand to realize our goal of controlling the conformation of G4 using light as an external stimulus. Upon irradiating a 10 μm solution of (*E*)‐**1** with 400 nm light in aqueous 100 mm Na^+^ phosphate buffer, the UV absorbance spectrum revealed significant changes.

Unlike for previously reported stiff‐stilbene derivatives, these changes were not consistent with *E*–*Z* isomerization. Specifically, the absence of isosbestic points and a marked decrease in absorbance at *λ*>325 nm indicated a different reaction pathway (Figure 4). Mass spectrometry analysis suggested photo‐oxidation as the dominant reaction pathway, identifying a species corresponding to ketone **6** and demethylated **7** (Figure S12). These products are expected to arise from the photo‐oxidation of (*E*)‐**1** in which addition of oxygen across the stilbene forms the *endo*‐peroxide **5**, which fragments in a manner previously reported for related systems (Scheme 2).^23^ Further evidence of photooxidation and demethylation was obtained by undertaking the photoreaction on a preparative scale and extracting the product from buffer for characterization by NMR spectroscopy. The evolution of a ketone was clearly visible (δ_C_=207 ppm), and the spectral data were in agreement with a pure sample of ketone **7** (Figures S13 and S14).

**Figure 4:**
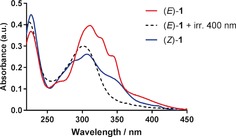
UV/Vis spectral changes in 10 μm (*E*)‐**1** upon irradiation with 400 nm blue light in 100 mm Na^+^ buffer. The spectrum of (*Z*)‐**1** is included for reference.

**Scheme 2:**
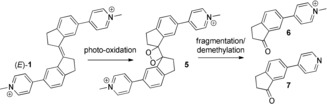
Proposed photoreaction pathway of (*E*)‐**1**.

We considered that this response of (*E*)‐**1** to blue light, coupled with the ability of the ligand to induce G4 unfolding, may allow the ligand to serve as a photoresponsive fuel for the regulation of G4. After first confirming that no changes to the CD spectrum of telo23 were caused by exposure to 400 nm light alone (Figure S15), telo23 G4 was exposed to (*E*)‐**1** (inducing the conformational switch, see above) and the solution was irradiated with 400 nm light for 20 min. Pleasingly, the CD spectrum acquired following irradiation exactly resembled the original spectrum of the G4 in the absence of the ligand (Figure 4 a, red trace), demonstrating that exposure to blue light deactivates (*E*)‐**1** and causes the G4 to switch back to the original topology.

To examine the reversibility of this process, we added a further aliquot of (*E*)‐**1** and recorded the CD spectrum again. As expected, the CD spectrum of ligand‐bound telo23 was restored almost completely (Figure 5 a, blue trace). The fuelling/irradiation process was repeated, and the conformation of the G4 was switched five times before significant fatigue of the system was observed (Figures 5 b and S16). We attribute this to dilution effects and an accumulation of the photo‐oxidation products. Further evidence for the reversible unfolding of G4 was obtained by NMR analysis (Figures 5 c and S9). The recovery and sharpening of the imino signals upon photoirradiation indicated the refolding of the DNA to regenerate the original G4. To the best of our knowledge (*E*)‐**1** is the first example of a G4 ligand reported to fuel the repeated light‐driven switching of telo23 folding in Na^+^‐rich conditions.

**Figure 5:**
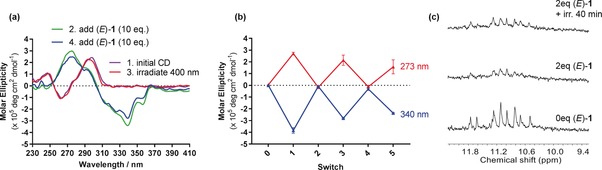
Reversible unfolding of telo23 in Na^+^ buffer by using (*E*)‐**1** as a photoresponsive fuel. a) CD spectra of telo23 generated by exposure to 400 nm light in the presence of (*E*)‐**1**. b) Plot of the change in ellipticity at 273 nm (red) and 340 nm (blue) over several dose/irradiation cycles. c) ^1^H NMR photoresponse sequence (imino section of the spectra).

In summary, we have identified a pyridinium stiff‐stilbene derivative as a novel G4 binding agent that displays high affinity for G4 DNA with significant discrimination against duplex DNA. The conformational switch induced in G4 by (*E*)‐**1**, coupled with the photochemistry of the underlying stiff‐stilbene scaffold, allows the reversible switching of topology in Na^+^ buffer over several cycles. (*E*)‐**1** and related derivatives therefore display exciting potential as supramolecular fuels for G4 nanomachines, allowing conformational regulation without the requirement to pre‐incorporate photoresponsive functionality into the biomolecule. Further investigations into the activity and application of stiff‐stilbene G4 ligands are already underway and will be reported in due course.

## Conflict of interest

The authors declare no conflict of interest.

## Supporting information

As a service to our authors and readers, this journal provides supporting information supplied by the authors. Such materials are peer reviewed and may be re‐organized for online delivery, but are not copy‐edited or typeset. Technical support issues arising from supporting information (other than missing files) should be addressed to the authors.

Supplementary
