# Supplementary material for: A Photoresponsive Stiff‐Stilbene Ligand Fuels the Reversible Unfolding of G‐Quadruplex DNA
Source: Angew Chem Int Ed Engl. 2019 Feb 20;58(13):4334–8. doi: 10.1002/anie.201900740 (PMC6563076; doi:10.1002/anie.201900740)
Supplement: Supplementary file 1 — Supplementary [file ANIE-58-4334-s001.pdf]

Angewandte Chemie
Eine Zeitschrift der Gesellschaft Deutscher Chemiker

Supporting Information

A Photoresponsive Stiff-Stilbene Ligand Fuels the Reversible Unfolding of G-Quadruplex DNA

*Michael P. O'Hagan, Susanta Haldar, Marta Duchi, Thomas A. A. Oliver, Adrian J. Mulholland, Juan C. Morales, and M. Carmen Galan**

anie_201900740_sm_miscellaneous_information.pdf

Contents

1 Experimental details 2
1.1 FRET melting assays 2
1.2 Circular dichroism titrations 4
1.3 Circular dichroism kinetic studies 4
1.4 UV-visible spectroscopy 5
1.5 NMR experiments 5
1.6 Photoirradiation experiments 5
2 Supplementary tables 6
3 Supplementary figures 7
4 Computational studies 17
5 Synthetic procedures and compound characterisation 22
6 NMR spectra of novel compounds 28
7 References 33

1 Experimental details

1.1 FRET melting assays

Fluorescence resonance energy transfer (FRET) melting assays were performed according to the procedure reported by De Cian and co-workers[1] on Roche LightCycler 480 qPCR instrument. In these assays, the oligonucleotides of interest were obtained labelled at the 5’ and 3’ ends with FAM (a fluorescence donor) and TAMRA (a fluorescence quencher) respectively. In the folded state, proximity of the donor and quencher mean that FAM fluorescence is not observed since energy is transferred non-radiatively to TAMRA by FRET. As the temperature is raised and the secondary structure denatures, the fluorophores move further apart and hence the fluorescence signal increases. From the resulting curve, the characteristic melting temperature ($T_{1/2}$) is defined as that at which the normalised fluorescence signal equals 0.5. The change in melting temperature (ΔT_m) induced by a small molecule ligand compared to that of the oligonucleotide in the absence of ligand provides an indication of the ligand’s ability to stabilise the G4 structure. The assay is shown in schematic form below:

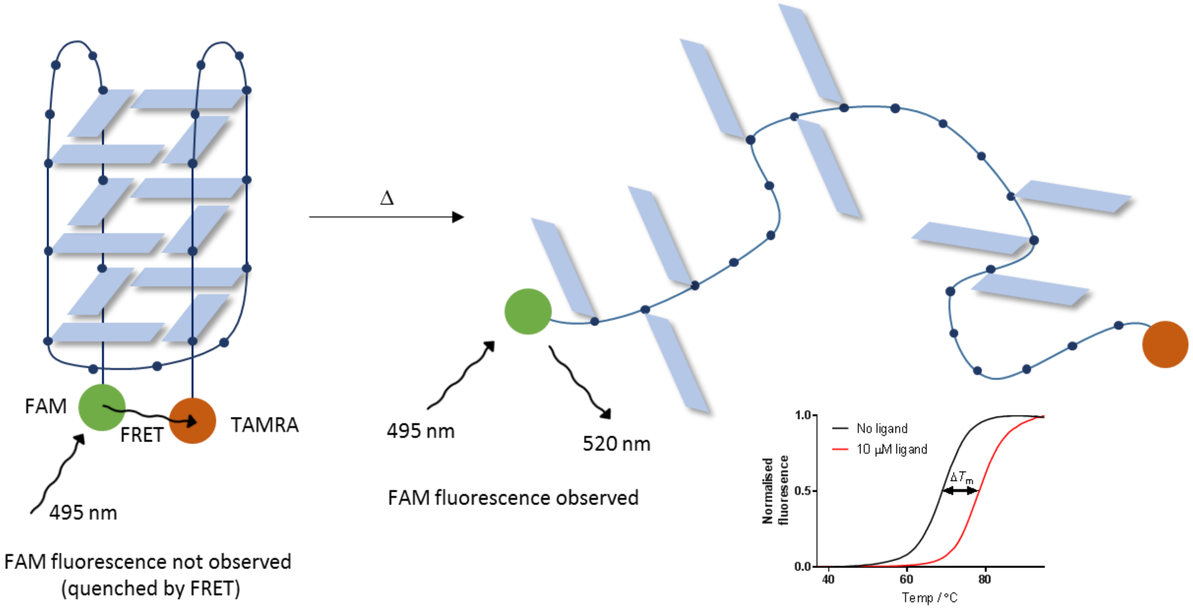

All oligonucleotides used were purchased from Eurogentec (Belgium), purified by HPLC and delivered dry. Oligonucleotide concentrations were determined by UV-absorbance using a NanoDrop 2000 Spectrophotometer from Thermo Scientific. The oligonucleotides used were:

**DNA model**	**Sequence**
F21T (human telomeric G4)	5'-FAM-GGGTTAGGGTTAGGGTTAGGG-TAMRA-3'
FmycT (c-myc promoter G4)	5'-FAM-TTGAGGGTGGGTAGGGTGGGTAA-TAMRA-3'
F10T (duplex)	5'-FAM-TATAGCTATA-HEG-TATAGCTATA-TAMRA-3'
ds26 (unlabelled duplex)	5'-CAATCGGATCGAATTCGATCCGATTG-3'

FAM = 6-carboxyfluorescein;
TAMRA = 6-carboxy-tetramethylrhodamine;
HEG = $[(-CH_2CH_2O-)_6]$

All sequences were annealed before use by heating for 2 minutes at 90°C and then placed immediately into ice. The final concentration of oligonucleotide was 200nM in all cases. The buffer used depended on the sequence in question, for F21T in Na^+ conditions, the final buffer contained 100mm NaCl, and 10 mm Li cacodylate. For FmycT (K^+ conditions), 1 mM KCl, 99 mM LiCl and 10 mM Li cacodylate were used. For F21T in K^+ conditions and F10T, 10 mM KCl, 90 mM LiCl and 10 mM Li cacodylate were used. Ligand concentrations were either 1 µM, 2 µM, 5 µM or 10 µM. Each sample was tested in duplicate on the same plate, and each experiment was tested in triplicate to assess the reproducibility of all results. Appropriate control experiments were also carried out for each sample set. Data processing was carried out using Origin 9, with $\Delta T_{1/2}$ used to represent ΔT_m.

1.2 Circular dichroism titrations

Circular Dichroism (CD) titrations were recorded using a Jasco J-810 spectrometer fitted with a Peltier temperature controller. Measurements were taken in a quartz cuvette with a path length of 5 mm, at 20 °C, at a 100 nm / min scanning speed at 1 nm intervals, with a 1 nm bandwidth. The CD spectra were recorded between 450 and 200 nm, and baseline corrected for the buffer used. The oligonucleotide sequence used was: telo23 (human telomeric G-quadruplex): 5'-TAGGGTTAGGGTTAGGGTTAGGG-3'. The oligonucleotide was purchased from Eurogentec (Belgium), purified by HPLC and delivered dry. Oligonucleotide concentrations were determined by UV-absorbance using a NanoDrop 2000 Spectrophotometer from Thermo Scientific. The oligonucleotide was annealed before use by heating for 2 minutes at 90°C and then placed immediately into ice. The oligonucleotide was at a concentration of 4.22 µM which gave an OD of 1 and the buffer used was either sodium or potassium phosphate (100 mM, pH 7.4). Oligonucleotide concentration remained constant throughout, and dilutions were made using a solution containing both oligonucleotide and the ligand, with the amount added determining the relative ratio of the two species. The reported spectrum for each sample represents the average of 3 scans. Data processing was carried out using Prism 7 with an 8-point second order smoothing polynomial applied to all spectra. Observed ellipticities were converted to mean residue ellipticity (θ) = deg cm^2 $dmol^{-1}$ (molar ellipticity).

1.3 Circular dichroism kinetic studies

Kinetic studies of the conformational switch were undertaken by monitoring the time course of the evolution of ellipticity at 273 nm at 20 °C following the addition of ligand. (*E*)-**1** (10 eq.) was added to the bottom of an empty cuvette (5 mm path length) in 4 µL DMSO. Following stabilisation of the background CD signal, telo23 (1 eq., 1 mL of 4.22 µM solution in 100 mM sodium phosphate buffer, pH 7.4) was injected rapidly to facilitate mixing and the change in CD monitored for 2500 sec. The addition point of ligand was visible by a spike in HT voltage and $t = 0$ defined as the moment this returned to a stable value (Figure S7b). Three independent repeats were conducted and the change in ellipticity at 273 nm plotted as a function of time. Fitting to a single exponential function ($\Delta\theta = A*\exp(-t/\tau) + c$) was carried out using Prism 7 to determine of the characteristic folding time (τ).

1.4 UV-visible spectroscopy

UV spectra were recorded on a Thermo Scientific BIOMATE 3S UV-vis Visible Spectrophotometer at ambient temperature. Measurements were taken in a quartz cuvette with a path length of 10 mm using in slow scanning mode at 0.5 nm intervals. The UV-visible spectra were recorded between 450 nm and 200 nm and baseline corrected for the buffer used.

1.5 NMR experiments

^{1}H NMR spectra of telo23 were recorded at 298 K using a 600 MHz Varian VNMRS spectrometer equipped with a triple resonance cryogenically cooled probe head (for imino NMR) or a 500 MHz Varian VNMRS spectrometer equipped with a broadband tuneable probe (for DOSY experiments). Samples of telo23 were dissolved in either 90% H_2O/10% D_2O (for imino NMR) or 100% D_2O (for DOSY experiments) containing 25 mM sodium phosphate (pH = 7.0) and 70 mM sodium chloride. All experiments employed sculpted excitation water suppression. DOSY experiments were conducted using the PGSE-based DOneshot sequence.[2] The final NMR samples contained 600 µL of 175 µM telo23 DNA. Samples were annealed before use by heating for 2 minutes at 90°C and then placed immediately into ice. Aliquots of (*E*)-**1** (10 mM in DMSO-d_6) were added to yield 2 equiv. portions and mixed thoroughly. NMR spectra were recorded immediately after the addition of ligand. Data were processed using MestReNova software (version 11.0.2).

1.6 Photoirradiation experiments

The 800 nm fundamental output of a Ti:Sapphire ultrafast amplifier (Libra, Coherent) was used to generate 397 nm via second harmonic generation for photoirradiation experiments. The spectral profile of the 397 nm light used is shown in Figure S11. The measured beam spot size (diameter) at the sample region is 0.4 cm and the energy used to irradiate solutions was 30 mW with a power density of 1.91 W cm^{-2}. Experiments involving (*E*)-**1** in the absence of telo23 were conducted using 1.5 mL solution in a 3 mL quartz cuvette with a path length of 10 mm. The concentration of the ligand was 10 µM in sodium phosphate buffer (100 mM, pH 7.4). The photoreaction was followed using UV-visible spectroscopy using the procedure detailed above (Section 1.4). Experiments involving telo23 were conducted using 1 mL solution in a 2 mL quartz cuvette with a path length of 5 mm. The concentration of DNA was 4.22 µM in sodium phosphate buffer (100 mm, pH 7.4). The effect of photoirradiation of G4 topology was

monitored by circular dichroism spectroscopy using the procedure detailed above (Section 1.2). For experiments involving the repeated addition of (*E*)-**1**, 10 eq. of ligand were added by aliquot (82 µL) of 600 µM (*E*)-**1** solution in water. The system was allowed to equilibrate for 20 min at 20 °C following addition of ligand before the CD spectrum was recorded. Photoirradiation of NMR samples was conducted by transferring the sample a 2 mL quartz cuvette with a path length of 5 mm.

2 Supplementary tables

Table S1: FRET stabilisation values for F21T (100 mM potassium cacodylate buffer, pH 7.4) by 1 µM (*E*)-**1** in the presence of increasing concentrations of competitor duplex sequence ds26.

Ratio ds26:F21T (base pairs:G4)	ΔT_m / °C
0:1 (0:1)	22.6 ± 0.7
2.5:1 (33:1)	21.5 ± 0.9
12.5:1 (163:1)	18.7 ± 0.8
25:1 (325:1)	16.5 ± 0.9

[Errors are reported as S.E.M]

Table S2: FRET stabilisation values for (*Z*)-**1**

ΔT_m / °C	1 µM	2 µM
F21T (K^+)	9.7 ± 0.5	12.6 ± 0.6
F21T (Na^+)	5.4 ± 1.0	7.3 ± 1.3
FmycT (K^+)	5.1 ± 1.4	10.3 ± 0.9
F10T (K^+)	0.1 ± 0.1	1.0 ± 0.1

[Errors are reported as S.E.M]

3 Supplementary figures

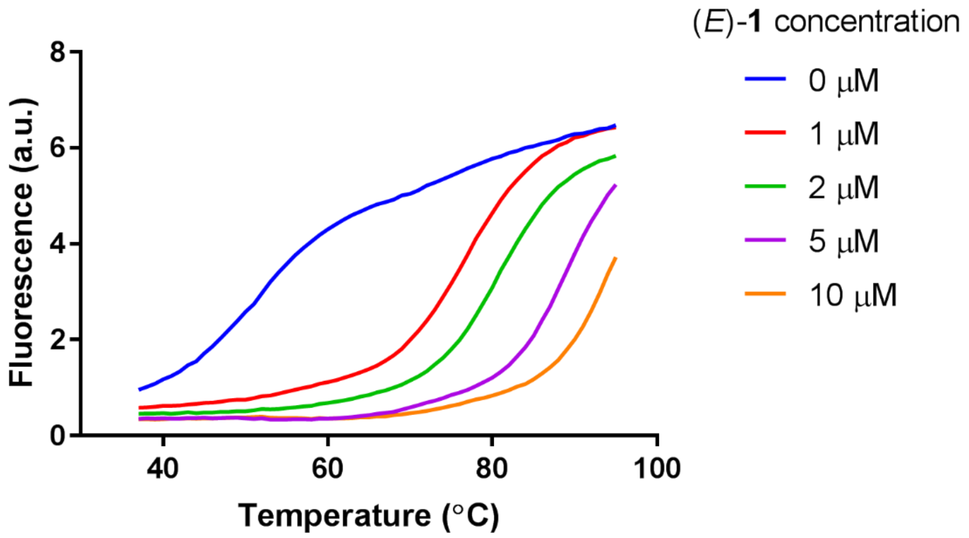

Figure S1: Representative thermal melting profiles for quadruplex sequence F21T (100 mM potassium cacodylate buffer, pH 7.4) in presence of increasing concentrations of *E*-(**1**) showing strong induced thermal stabilisation. At higher concentrations (5 μM and 10 μM) the induced thermal stabilisation is too high to extract meaningful ΔT_m values as the G4 remains appreciably folded at the maximum temperature.

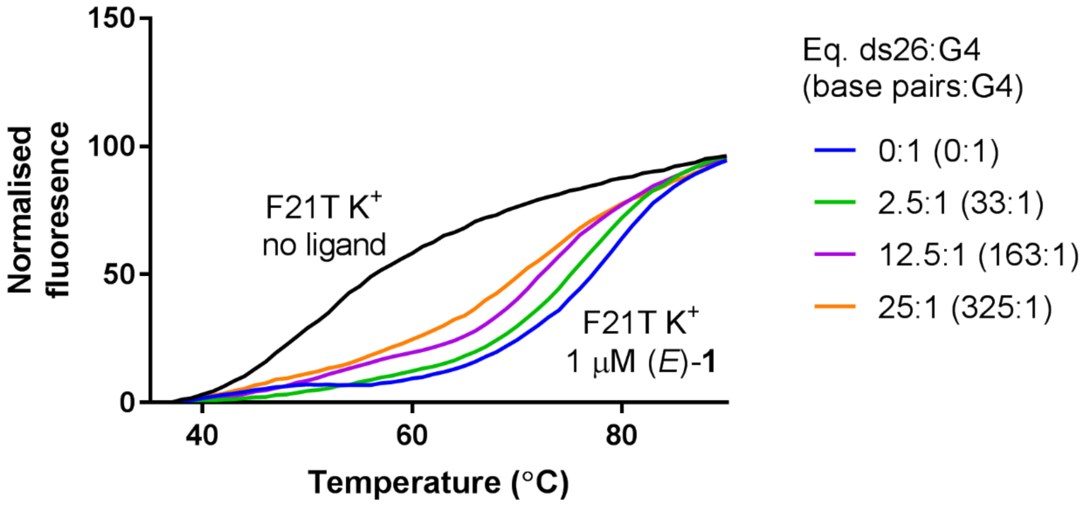

Figure S2: Representative thermal melting profiles for quadruplex sequence F21T (100 mM potassium cacodylate buffer, pH 7.4) in the presence of 1 μM (*E*)-**1** and increasing equivalents of competitor duplex ds26. The minimal effect on the melting profile by ds26 reflects the high specificity of (*E*)-**1** for the four-stranded G4 structure.

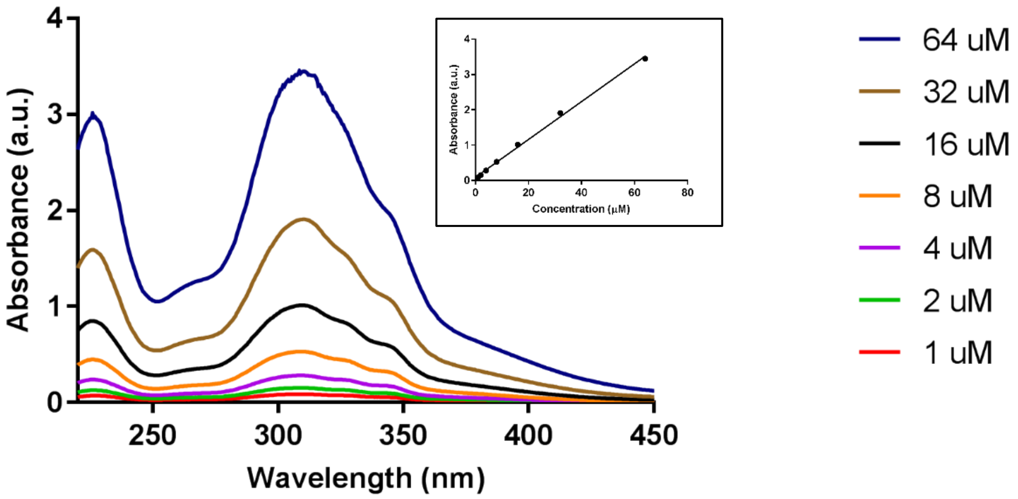

Figure S3: Concentration dependence of (*E*)-**1** UV-vis absorbance. Inset shows linear relationship in accordance with the Beer-Lambert law.

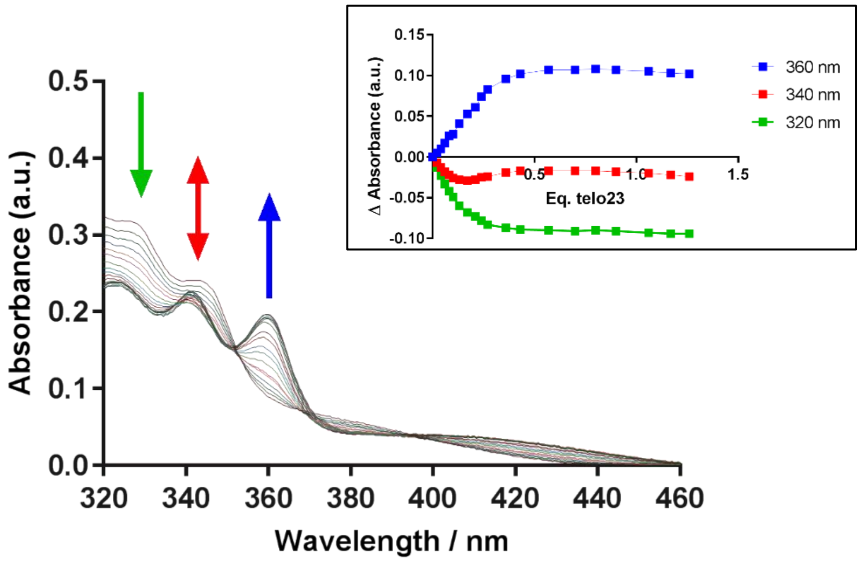

Figure S4: UV-visible titration of 10 μM (*E*)-**1** with telo23 in K^+ buffer. Only regions outside of DNA absorbance (λ > 320nm) are shown. The isotherms were not described well by 1:1 or 2:1 binding models, precluding accurate determination of the association constant.

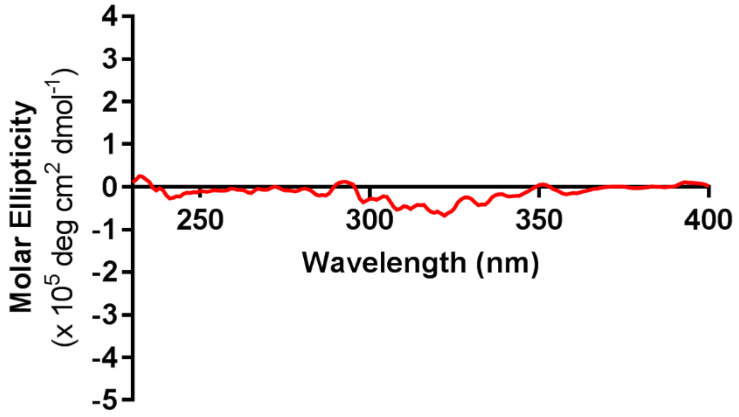

Figure S5: CD spectra of (*E*)-**1** (75 µM) in the absence of telo23 DNA (100 mM sodium phosphate buffer, pH 7.4).

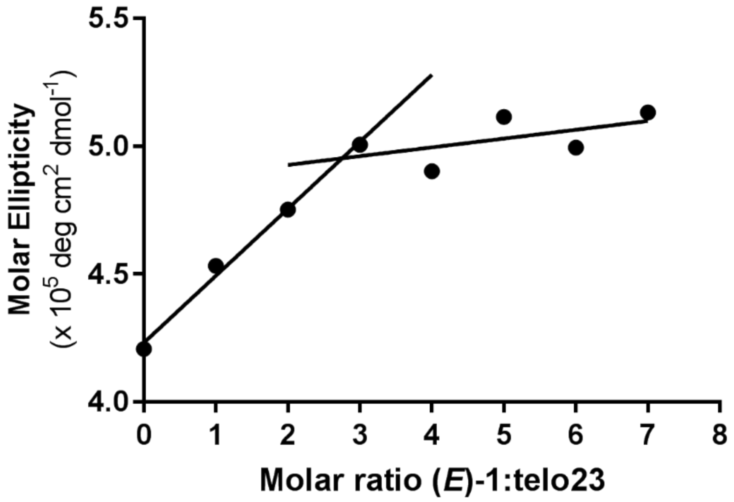

Figure S6: Change in ellipticity at 288nm of telo23 in K^+ buffer on titration with (*E*)-**1.** The inflection point suggests a 3:1 stoichiometry for the (*E*)-**1**:telo23 complex.

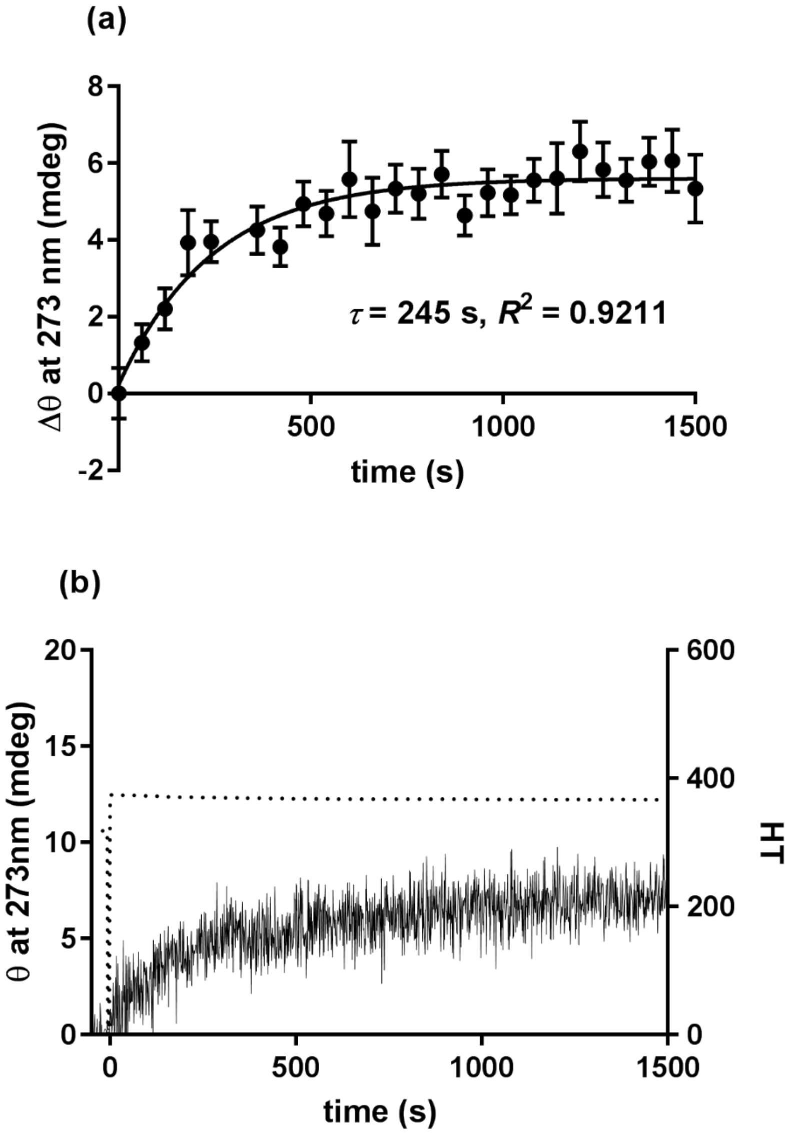

Figure S7: (a) Kinetic profile showing change in ellipticity at 273 nm following mixing of telo23 (4.22 μM) and 10 eq. (*E*)-**1** in 100 mM sodium phosphate buffer (pH = 7.4). The error bars show the standard deviation of three repeats. Data are fitted to a single exponential function $\Delta\theta = A*\exp(-t/\tau) + c$. (b) Example raw data from a circular dichroism kinetic study. $t = 0$ is defined as the point where the HT voltage (dotted line) returns to its maximum value following the negative spike observed on addition of oligonucleotide solution.

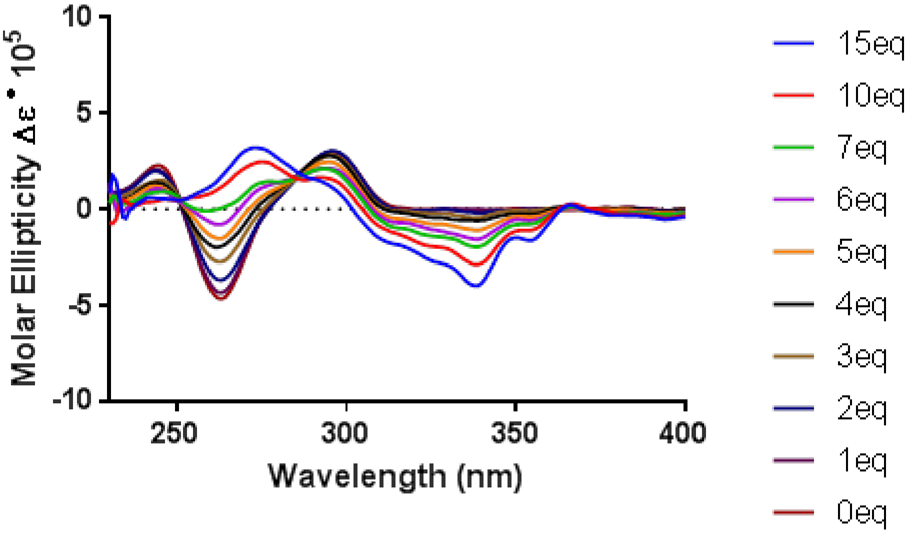

Figure S8: Circular dichroism titration of (*E*)-**1** into antiparallel telo22 in 100mM Na^{+} phosphate buffer, pH = 7.4.

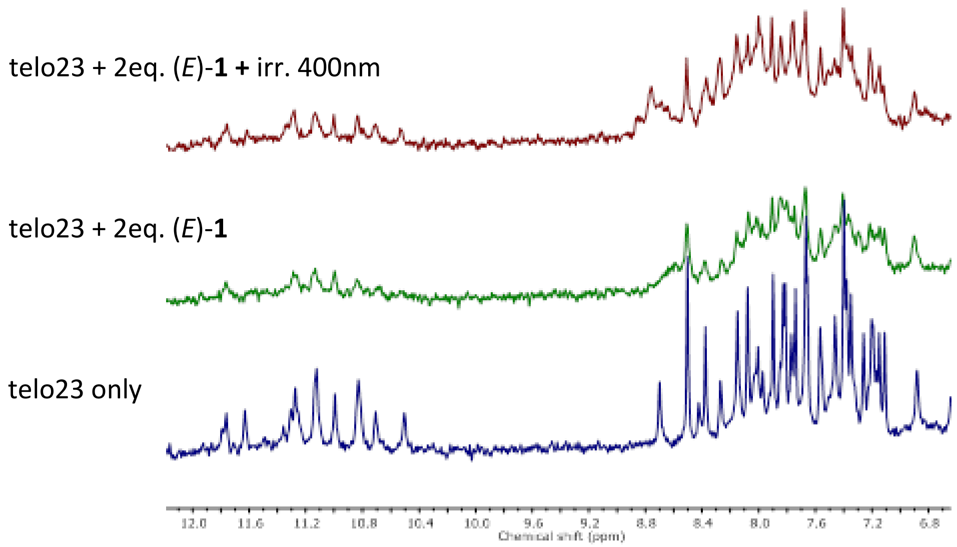

Figure S9: Imino and aromatic region of telo23 spectra (175 µM DNA in sodium phosphate buffer) in the absence of (*E*)-**1** (lower), in the presence of 2 eq. (*E*)-**1** (middle) and after photoirradiation (upper). Recovery of imino signals and sharpening of aromatic signals is observed upon photoirradiaton.

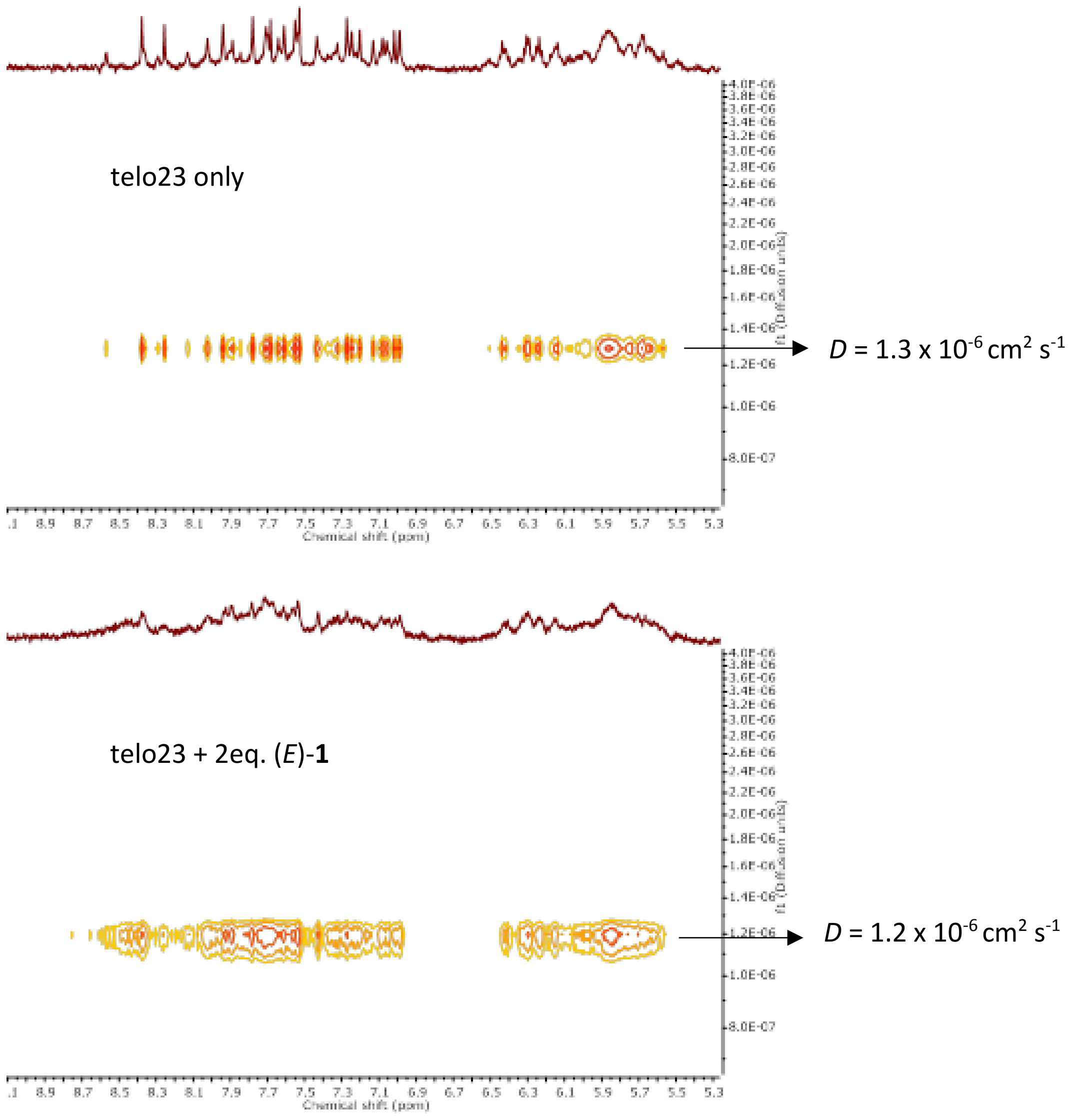

Figure S10: DOSY spectra and diffusion coefficients of telo23 (175 μM DNA in sodium phosphate buffer) in the absence (upper) and presence (lower) of 2 equivalents of (*E*)-**1**.

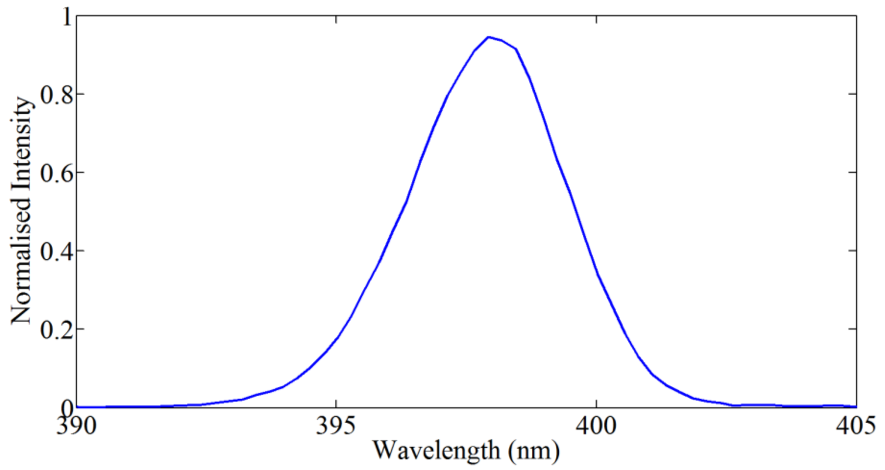

Figure S11: Spectral profile of the 30mW beam used to irradiate solutions of (*E*)-**1.**

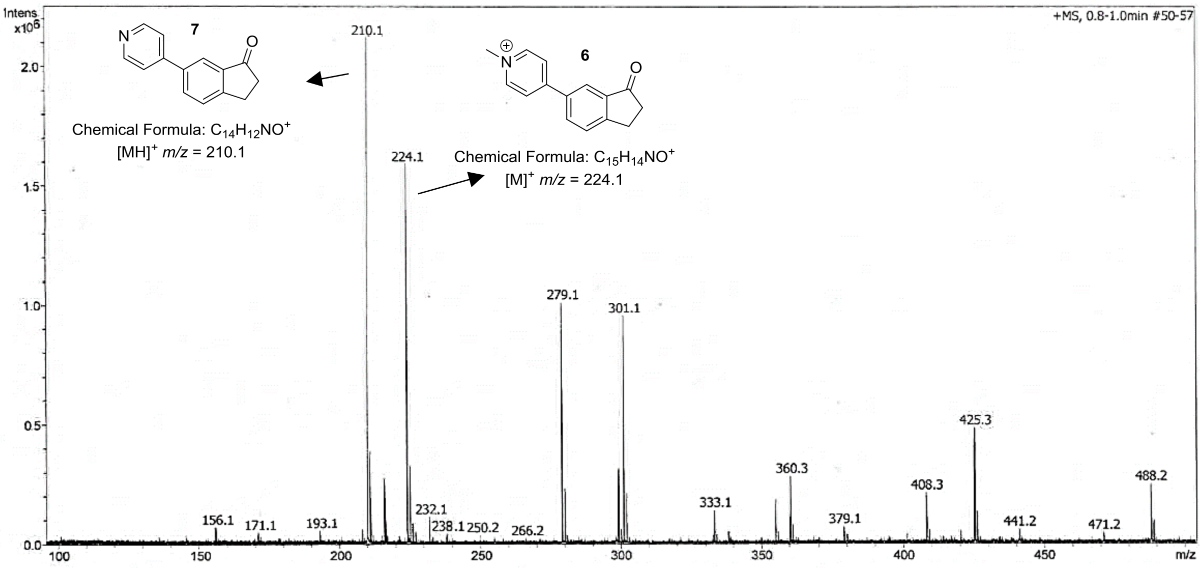

Figure S12: ESI-MS of 10 μM (*E*)-**1** following 90 min photoirradiation in 100 mM sodium phosphate buffer (pH = 7.4).

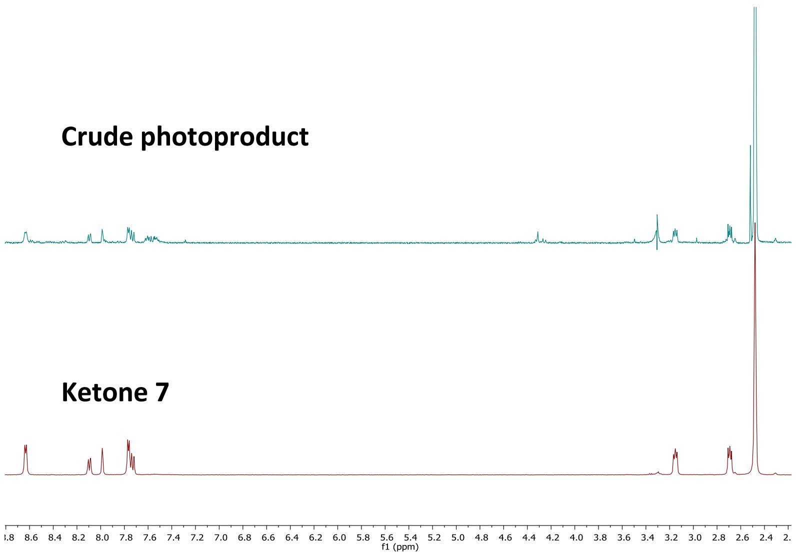

Figure S13: Comparison of ^{1}H NMR spectra of crude photoproduct and ketone **7**

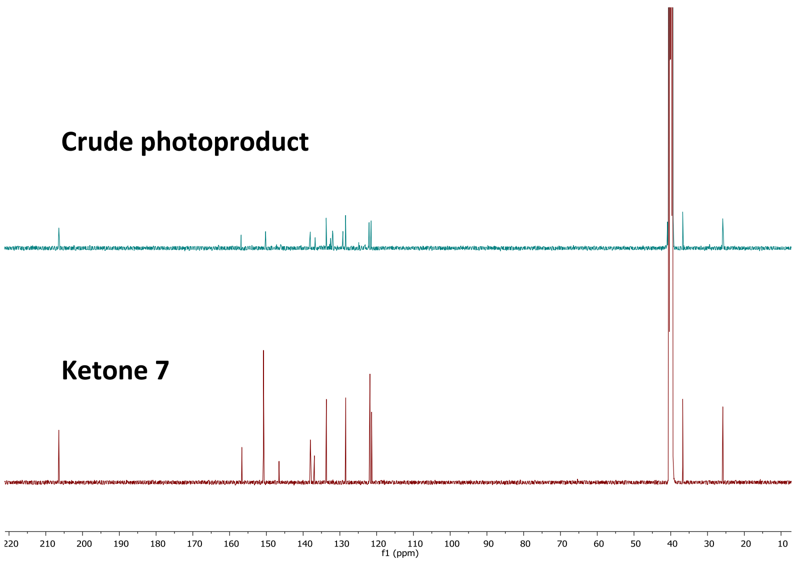

Figure S14: Comparison of ^{13}C NMR spectra of crude photoproduct and ketone **7**

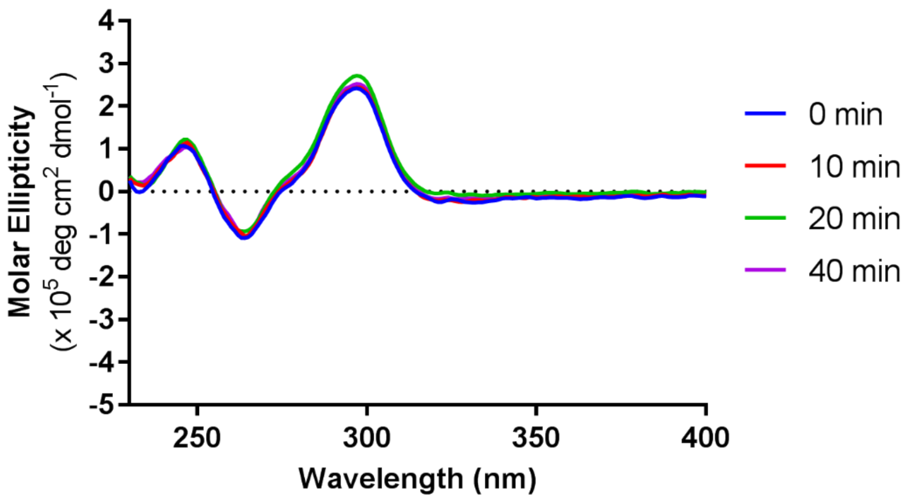

Figure S15: CD spectra of telo23 G4 before and after 400 nm irradiation in absence of (*E*)-**1**

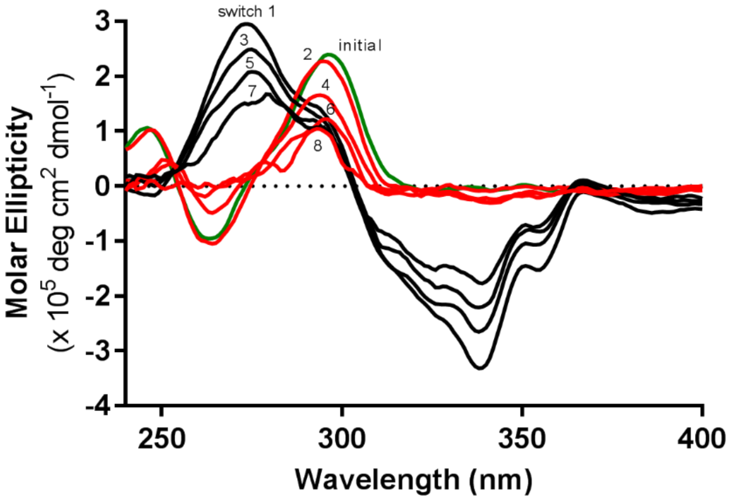

Figure S16: Eventual photofatigue of the (*E*)-**1**/telo23 conformational switch showing spectra initial spectrum (green), spectra following addition 10 eq. portions of (*E*)-**1** (black; switches 1, 3, 5, and 7) and spectra following photoirradiation (red; switches 2, 4, 6 and 8). Though a topological switch can be clearly observed over the 8 cycles, little recovery of the negative band at 265 nm and over 50% reduction in the maximum at 295 nm is observed following switch 5.

4 Computational studies

We performed a docking calculation of (*E*)-**1** to telo22 G4 DNA (PDB code: 143D)[3] in order to predict all the available high-affinity binding modes. The ligand molecule was optimised using PerkinElmer Chem3D software using the MM2 forcefield. Docking calculations were performed using AutoDock Vina[4] with the DNA structure kept fixed in its original crystal conformation throughout the docking procedures. The highest affinity binding pose was then submitted to long microsecond molecular dynamics simulations in order to predict the stability of the binding pose.

Standard (unbiased) molecular dynamics simulations

All the simulations were performed using the Grgmacs-5.0 software package.[5] The recently introduced parm-BSC1 force field was used for the DNA parameterization. For the ligand, the General Amber Force Field (GAFF) were used to generate parameters.[6,7] The charges were calculated using the restrained electrostatic potential (RESP) fitting procedure.[8] The RESP fit was performed onto a grid of electrostatic potential points calculated at the HF/6-31G(d) level as recommended by the force-field designers and recent literature.[7,8]

To start the simulations, each docked (*E*)-**1**/telo22 complex was solvated in a cubic box with the dimension of 74 x 74 x 74 $Å^3$ along with 12982 TIP3P explicit water molecules.[9] An extra 19 Na+ ions were added to neutralize the system.[10] The (*E*)-**1**/telo22 complexes were minimized prior to the equilibration and production run as follows: the minimization of the solute hydrogen atoms on the DNA and the ligand was followed by the minimization of the counterions and the water molecules in the box. In the next step, the DNA backbone along with the all the heavy atoms on the ligand were kept frozen, and the solvent molecules with counterions were allowed to move during a 50 ps MD run, to relax the density of the whole system. In the next step the nucleobases were relaxed in several minimization runs with decreasing force constants applied to the DNA backbone atoms, however, a few phosphate atoms were kept restrained with a force constant of 2.39 kcal.mol-1 . $Å^{-2}$. After the full relaxation, the system was slowly heated to the room temperature to 300K using V-rescale thermostat with a coupling constant of 0.5 ps employing an NVT (constant-temperature, constant-volume) ensemble.[11,12] As the system reached the temperature of interest, the equilibration simulation was performed for 10000 ps (10 ns) using an NPT ensemble with

Berendsen thermostat and Berendsen barostat, and 0.5 ps was used again as the coupling constant for both temperature and pressure, respectively.[13] Finally, the production run was set for 1000000000 ps (1μs) using Nose-Hoover thermostat[14,15] and Parrinello-Rahman barostat[16] with the same coupling constant as previously taken in the equilibration simulation in the NPT ensemble. All the simulations were carried out under the periodic boundary conditions (PBC). The particle-mesh Ewald (PME) method was used to calculate the electrostatic interactions with in a cut-off of 10 Å.[17] The same cut-off was used for Lennard-Jones (LJ) interactions. All simulations were performed with a 1.0 fs time step.

Well-tempered metadynamics

We performed a well-tempered metadynamics (WTMetaD) simulation of (*E*)-**1** binding to the telo22 G4 DNA.[18] The WTMetaD also helps to understand the binding/unbinding mechanism of (*E*)-**1**. The simulation started with a well-equilibrated structure generated from the docking pose-1 (*vide infra*). As discussed above, the docking poses had been run for 1 μs in MD simulations; however, the preliminary structure for the WTMetaD simulation was taken after 20 ns of MD simulation, which was found to be a stable ligand binding conformation.
The same systems and MD settings as described previously were used for the WTMetaD simulation. The plumed 2.3 plugin was used to carry out the simulation with the Gromacs-5.0.7 code.[19] The bias potential was calculated according to the WTMetaD scheme as follows:

$$V(s,t) = \sum_{t'=0,\tau_G,2\tau_G,\dots}^{t'<t} \omega\tau_G \, e^{-V(s(q(t'),t'))/\Delta T} \, e^{-\sum_{i=1}^{2}[(s_i(q)-s_i(q(t')))^2/2\sigma_i^2]} \quad (1)$$

where the deposition rate, ω, and deposition stride, τG, of the Gaussian hills were set to 0.358 kcal.mol-1·ps-1 (1.5 kJ.mol-1·ps-1) and 1.0 ps, respectively. The bias factor (T + ΔT)/T was set to 15, and the final free energy surface (FES) was calculated as follows:

$$F(s,t) = -\frac{T+\Delta T}{\Delta T}(V(s,t) - C(T)) \quad (2)$$

where the V(s,t) is the bias potential added to the Collective Variables (CV) used and the T represents the simulation temperature. ΔT is the difference between the temperature of the CV and the simulation temperature. The bias potential is grown as the sum of the Gaussian hills deposited along the chosen CV space and finally the sampling of particular CV space can be controlled with the tuning of the ΔT parameter.

To describe the different ligand binding conformations during the metadynamics simulation, we used two Collective Variables (CV) and they are: (i) the distance (d) between the centre of mass (COM) of the middle G-tetrads and the heavy atoms of (*E*)-**1** and (ii) a torsion angle is used. The torsion is defined between two points in the ligand to two points in the G-tetrad. In the ligand, the two points are considered as the COM of the two indane residues.

MD analysis results

We carried out 1 μs long MD simulation of the lowest-energy docking pose. Figure S17 depicts all binding conformations sampled and the overall backbone RMSD of the telo22 G4 DNA. In order to analyse the possible sampling conformations, we checked the dihedral distribution where the same dihedral is used as taken in the WTMetaD simulation. Three stable peaks (A, B and C) are observed which corresponds to three successive conformations sampled by the binding pose. In all the cases, (*E*)-**1** is always seen to be located top of the DNA, making stacking interactions with A7, A19 and partially interacting with the G-tetrad (B). The overall RMSD of the DNA is found to be constant at ~3Å.

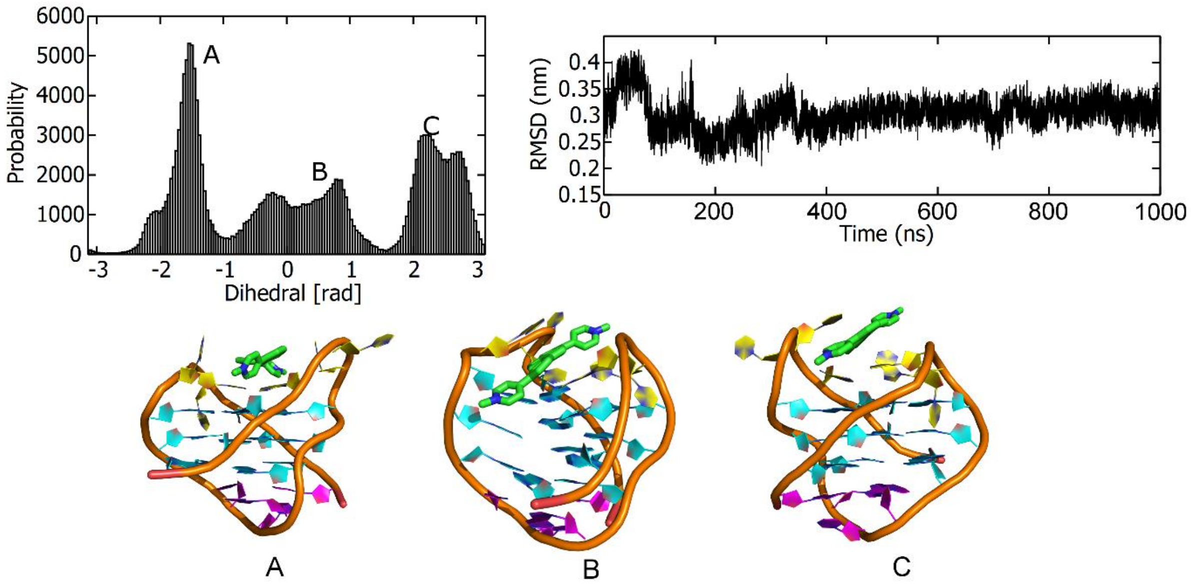

Figure S17: (upper left) dihedral distribution of conformations sampled; (upper right) backbone RMSD of telo22 DNA; (below) the three possible conformations sampled in 1 μs long MD simulation corresponding to the maxima A, B, C in the dihedral distribution,

Metadynamics results

The sampling of the ligand binding/unbinding mechanism was studied using the WTMetaD simulation. The simulation started with a well-equilibrated structure from the docking pose discussed above. As the simulation progressed, several binding/rebinding events were observed. Figure S18 depicts the separation distance between the centre of mass (COM) of the bases containing the central G-tetrad of telo22 i.e., the COM of G3, G9, G15, and G21 to the COM of the heavy atoms of the ligand (*E*)-**1**. Carefully looking at the distance separation and molecular visualisation, it is apparent that the ligand unbinds from DNA within the first ~15 ns and rapidly starts sampling the unbound state, i.e. the solvated state. Before the unbinding, the ligand visits the major groove and slides on top of the DNA interacting with A7 and A19 bases with stacking interactions (pose B in Figure S19). While sampling in this particular state, the ligand tail is partially interacting with thymine bases such as T5, T18 and T18 which are present on top of the DNA and always fraying toward solvent (pose C in Figure S19). As the time progresses, the separation distance increases and the ligand lose interactions with bases A7 and A19. At the final stage of unbinding, the ligand is interacting with T17 and T18 bases and leaves toward solvent (pose D in Figure S19). After the complete dissociation, the ligand mostly samples the unbound state and partially interacts with all the solvent exposed thymine bases until approximately 90 ns, where it enters to the major groove. During this process, all three G-tetrads are partially broken with rapid conformational change in the DNA which helps the ligand to partially intercalate (pose E in Figure S19). The 1st intercalation takes place at ~95 ns, making stacking interactions with G9 base which remain after ~10 ns of rigorous sampling. In the later stage of the simulation, the ligand returns towards G9 base and finally a full intercalation takes place at ~150 ns (pose F in Figure S19). At this stage of the simulation, the ligand mainly interacts with the guanine bases in the G-tetrads such as G3, G4, G9, G21 and G20, and also with A7 in a sandwich manner. After sampling for a further ~35 ns, the ligand leaves the DNA and again starts sampling the solvent region. In the meantime, several binding/unbinding processes is occurred (see Figure S18, the sampling region between ~350−525ns) and the DNA backbone RMSD is already reached to ~1.0 nm which corresponds towards the denaturation of the telo22 G4 structure. At ~525 ns, the ligand again enters to the binding pocket and intercalates with G21, G8, G15, G2 and G14 (see pose G in Figure S19). Finally, at ~582 ns, the telo22 G4 begins to unfold, with the backbone RMSD reaching ~1.5nm within the next 100 ns. These findings (see main manuscript) lead us to conclude that ligand (*E*)-**1** is able to unfold the telo22 G4 DNA upon binding thorough rapid major groove binding and intercalation, reinforcing our experimental observations.

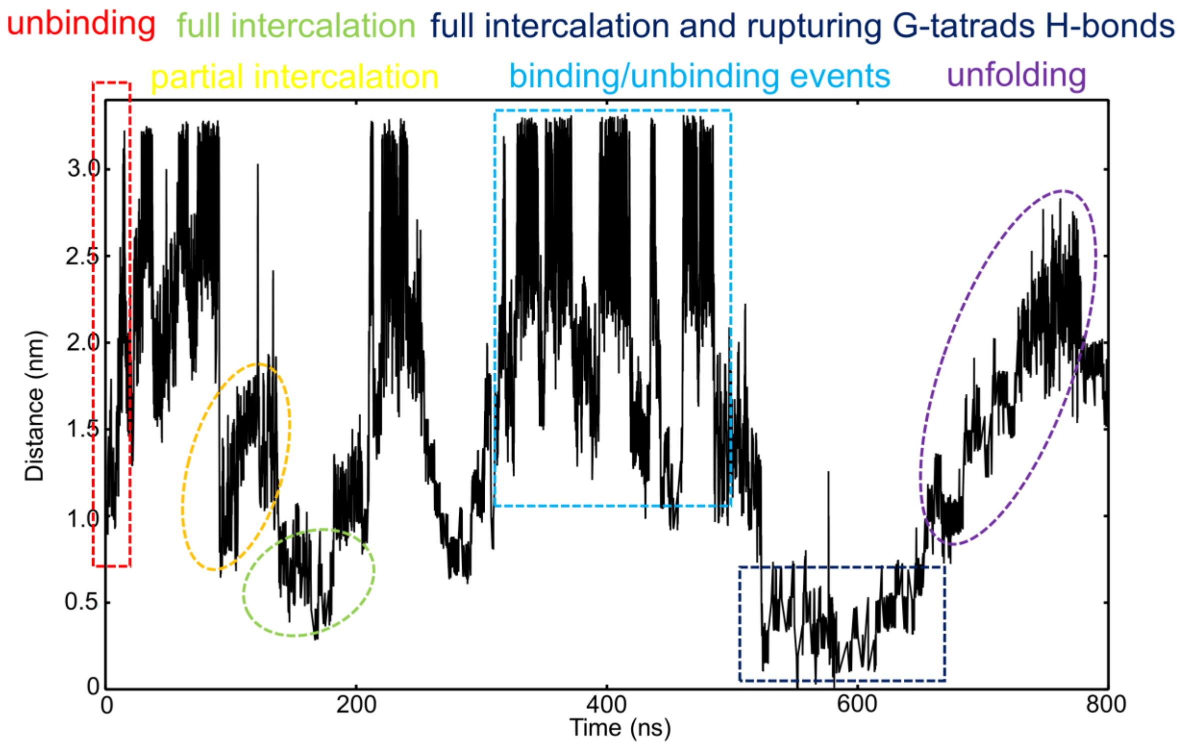

Figure S18: The separation distance between telo22 and (*E*)-**1** in the WTMetaD simulation. The highlighted sections in the curve represents all the possible events on the unfolding of telo22 DNA and each successive events is denoted with the corresponding colour codes.

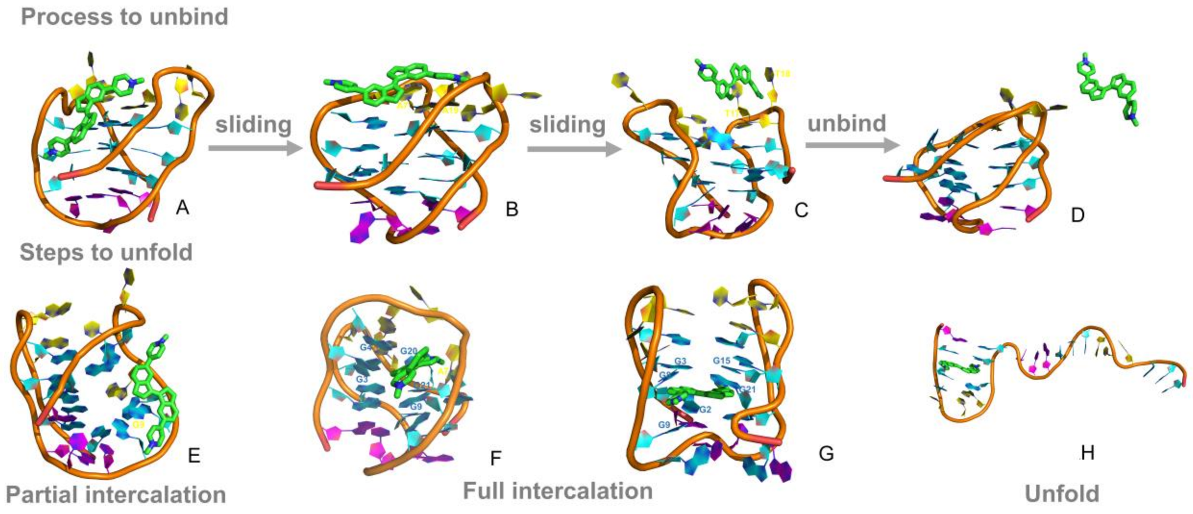

Figure S19: The schematic representation of the unbinding of (*E*)-**1** and the unfolding of telo22 DNA sampled in the WTMetaD simulation.

5 Synthetic procedures and compound characterisation

General experimental

Chemicals were purchased and used without further purification. Dry solvents were obtained by distillation using standard procedures, or by passage through a column of anhydrous alumina using equipment from Anhydrous Engineering (University of Bristol) based on the Grubbs' design.[20] Reactions requiring anhydrous conditions were performed under N_2; glassware and needles were either flame dried immediately prior to use, or placed in an oven (150 °C) for at least 2 h and allowed to cool in a desiccator or under reduced pressure. Liquid reagents, solutions or solvents were added via syringe through rubber septa; solid reagents were added via Schlenk type adapters. Reactions were monitored by TLC on Kieselgel $60F_{254}$ (Merck), with UV light (254 nm) detection and by staining with basic potassium permanganate solution. Flash column chromatography was performed according to Still and co-workers,[21] using silica gel [Merck, 230−400 mesh (40−63 µm)]. Solvents for flash column chromatography (FCC) and thin layer chromatography (TLC) are listed in volume:volume percentages. Extracts were concentrated *in vacuo* using both a Heidolph HeiVAP Advantage rotary evaporator (bath temperatures up to 50 °C) at a pressure of 15 mmHg (diaphragm pump) or 0.1 mmHg (oil pump), as appropriate, and a high vacuum line at room temperature. Water soluble compounds were freeze dried on a Lytotrap Plus (LTE Scientific LTD). ^{1}H NMR and ^{13}C NMR spectra were measured at 25°C in the solvent specified with Varian or Bruker spectrometers operating at field strengths listed. Chemical shifts are quoted in parts per million with spectra referenced to the residual solvent peaks. Multiplicities are abbreviated as: br (broad), s (singlet), d (doublet), t (triplet), q (quartet), p (pentet), m (multiplet) and app. (apparent) or combinations thereof. Assignments of ^{1}H NMR and ^{13}C NMR signals were made where possible, using COSY, HSQC and HMBC experiments. Mass spectra were obtained by the University of Bristol mass spectrometry service by electrospray ionisation (ESI) or matrix assisted laser desorption ionisation (MALDI) modes. Infra-red spectra were recorded in the range 4000-400 cm^{-1} on a Perkin Elmer Spectrum either as neat films or solids compressed onto a diamond window.

(*E*)-6,6'-dibromo-2,2',3,3'-tetrahydro-1,1'-biindenylidene, (*E*)-3

(*Z*)-6,6'-dibromo-2,2',3,3'-tetrahydro-1,1'-biindenylidene, (*Z*)-3

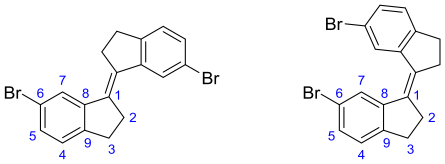

Zinc powder (5.898 g, 90.21 mmol) was suspended in anhydrous THF (75 mL). $TiCl_4$ (4.9 mL, 45 mmol) was added dropwise as the solution was stirred vigorously. The solution was heated to reflux and stirred for 2 h. The mixture was then cooled to rt and 6-bromo-1-indanone (4.760 g, 22.55 mmol) was added to the suspension. The mixture was heated to reflux and stirred for 16 h, then quenched with sat. aq. NH_4Cl solution (50 mL) and extracted with $CHCl_3$ (3 × 100 mL). The combined organic extractions were washed with H_2O (100 mL) and then concentrated *in vacuo* to approximately 30 mL, at which volume a precipitate formed. The precipitate was collected by filtration under reduced pressure and dried under vacuum to afford bromide (*E*)-**3** as an off-white solid (2.442 mg, 55%). The filtrate was concentrated *in vacuo* and the residue purified by flash silica chromatography, eluting with hexane, to afford bromide (*Z*)-**3** as a white solid (1.003 mg, 22%).

(*E*)-**3**:

^{1}H NMR (500 MHz, DMSO-d$_6$) δ 7.68 (2H, d, *J* = 1.8 Hz, 7-C*H*), 7.41 (2H, dd, *J* = 8.0, 1.8 Hz, 5-C*H*), 7.33 (2H, d, *J* = 8.0 Hz, 4-C*H*), 3.13 – 3.08 (4H, m, 2-CH_2 or 3-CH_2), 3.07 – 3.02 (4H, m, 2-CH_2 or 3-CH_2).

^{13}C NMR Too insoluble for this experiment.

ν_{max} / cm^{-1} (compressed solid) 2925 (w), 2887 (w), 2850 (w), 1889 (w), 1589 (m), 1560 (m), 1464 (m), 1433 (m), 1405 (m), 1205 (m), 1174 (m) 1078 (m), 1037 (m), 879 (s), 819 (s).

EI-LRMS for $C_{18}H_{14}Br_2^+$ $[M]^+$ calculated: 389.9, found: 390.2.

Proton NMR was consistent with literature data.[22]

(*Z*)-**3**:

^{1}H NMR (500 MHz, DMSO-d$_6$) δ 8.01 (2H, d, *J* = 1.8 Hz, 7-C*H*), 7.39 (2H, dd, *J* = 8.0, 1.8 Hz, 5-C*H*), 7.32 (2H, d, *J* = 8.0 Hz, 4-C*H*), 2.93 – 2.90 (4H, m, 2-CH_2 or 3-CH_2), 2.80 – 2.76 (4H, m, 2-CH_2 or 3-CH_2).

^{13}C NMR (126 MHz, DMSO-d$_6$) δ 147.6 (9-*C*), 141.8 (8-*C*), 135.2 (1-*C*), 130.2 (7-*C*H), 127.6, 125.0 (4-*C*H and 5-*C*H), 118.7 (6-*C*), 34.4 (2-*C*H or 3-*C*H), 29.6 (2-*C*H or 3-*C*H).

ν_{max} / cm^{-1} (compressed solid) 2029 (w), 2895 (w), 2871 (w), 2837 (w), 1635 (w), 1590 (m), 1559 (m), 1459 (m), 1444 (m), 1425 (m), 1264 (m), 1166 (m), 1071 (m), 803 (s).

EI-LRMS for $C_{18}H_{14}Br_2^+$ $[M]^+$ calculated: 389.9, found: 390.0.

Proton and carbon NMR were consistent with literature data.[22]

(*E*)-6,6'-di(pyridin-4-yl)-2,2',3,3'-tetrahydro-1,1'-biindenylidene, (*E*)-4

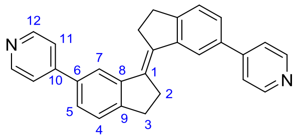

Bromide (*E*)-**3** (245 mg, 0.628 mmol) and 4-pyridinylboronic acid (232 mg, 1.88 mmol) dissolved in 9:1 toluene:EtOH (25 mL) and 2M aq. Na_2CO_3 (5 mL) was added. The solution was degassed by bubbling nitrogen for 30 min. $Pd(PPh_3)_4$ (144 mg, 0.123 mmol) was added in one portion and the mixture degassed for a further 10 min. The reaction was heated to 100 °C and stirred for 16 h. The mixture was then cooled to rt, diluted with CH_2Cl_2 (150 mL) and washed with H_2O (100 mL). The aqueous was back extracted with CH_2Cl_2 (50 mL) and the combined organic extractions dried ($MgSO_4$) and filtered. The filtrate was concentrated *in vacuo* and the residue purified by flash silica chromatography, eluting with 0 – 5% MeOH in CH_2Cl_2. The resultant solid was triturated in Et_2O (30 mL), filtered and air-dried to afford the title compound as an off-white powder (128 mg, 53%).

^{1}H NMR (500 MHz, $CDCl_3$) δ 8.68 (4H, d, *J* = 6.2 Hz, 12-C*H*), 7.86 (2H, s, 7-C*H*), 7.56 (4H, d, *J* = 6.2 Hz, 11-C*H*), 7.50 (2H, d, *J* = 7.8 Hz, 5-C*H*), 7.46 (2H, d, *J* = 7.8 Hz, 4-C*H*), 3.34 – 3.29 (4H, br, 2-C*H*$_2$), 3.24 – 3.20 (4H, br, 3-C*H*$_2$).

^{13}C NMR (126 MHz, $CDCl_3$) δ 150.4 (12-*C*H), 149.1 (10-*C*), 148.5 (9-*C*), 144.2 (8-*C*), 136.9 (6-*C*), 135.9 (1-*C*), 126.3 (5-*C*H), 125.8 (4-*C*H), 123.2 (7-*C*H), 121.9 (11-*C*H), 32.4 (2-*C*H$_2$), 31.1 (3-*C*H$_2$).

ν_{max} / cm^{-1} (compressed solid) 3025 (w) 2942 (w), 2929 (w), 2911 (w), 2845 (w), 1593 (s), 1542 (m), 1474 (s), 1453 (w) 1434 (w), 1422 (w), 1440 (m), 1270 (m), 1069 (m), 992 (m), 887 (w), 839 (m), 812 (s), 800 (s), 729 (m), 607 (m), 535 (s).

ESI-HRMS for $C_{28}H_{23}N_2^+$ $[M+H]^+$ calcd: 387.1856, found: 387.1871.

(*E*)-4,4'-(2,2',3,3'-tetrahydro-[1,1'-biindenylidene]-6,6'-diyl)bis(1-methylpyridin-1-ium) iodide, (*E*)-1

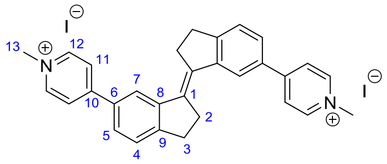

Pyridine (*E*)-**4** (20 mg, 0.52 mmol), was dissolved in anhydrous DMF (2 mL) and methyl iodide (60 µL, 0.96 mmol) was added. The reaction was heated to 90 °C and stirred for 16 h. The mixture was then cooled to rt and concentrated *in vacuo.* The residue was suspended acetone (5 mL), filtered and the dark solid air dried to afford the title compound (14 mg, 41%).

^{1}H NMR (500 MHz, DMSO-d_6) δ 9.00 (4H, d, *J* = 6.9 Hz, 12-C*H*), 8.56 (4H, d, *J* = 6.9 Hz, 11-C*H*), 8.11 (2H, d, *J* = 1.6 Hz, 7-C*H*), 7.95 (2H, dd, *J* = 7.9, 1.6 Hz, 5-C*H*), 7.66 (2H, d, *J* = 7.9 Hz, 4-C*H*), 4.36 (6H, s, 13-CH_3), 3.40 – 3.36 (4H, m, 2-CH_2), 3.25 – 3.22 (4H, m, 3-CH_2).

^{13}C NMR (126 MHz, DMSO-d_6) δ 154.8 (10-*C*), 151.6 (9-*C*), 145.5 (12-*C*H), 143.8 (8-*C*), 135.7 (1-*C*), 132.4 (6-*C*H_2), 127.6 (5-*C*H), 126.4 (4-*C*H), 124.3 (11-*C*H), 123.6 (7-*C*H), 47.1 (13-*C*H_3), 31.4 (2-*C*H_2), 30.7 (3-*C*H_2).

ν_{max} / cm^{-1} (compressed solid) 3121 (w), 3088 (w), 3038 (w), 2930 (w), 2910 (w), 2844 (w), 1636 (s), 1601 (s), 1558 (m), 1522 (m), 1472 (s), 1439 (m), 1414 (m), 1348 (w), 1312 (m) 1298 (m), 1289 (m), 1272 (m), 1223 (w), 1198 (s), 856 (w), 820 (s), 800 (m), 723 (m), 607 (m), 587 (m), 534 (m), 510 (s), 426 (s).

MALDI-HRMS $C_{29}H_{25}N_2^+$ $[M-CH_3]^+$ calculated: 401.2012, found 401.2019.

(*Z*)-6,6'-di(pyridin-4-yl)-2,2',3,3'-tetrahydro-1,1'-biindenylidene, (*Z*)-4

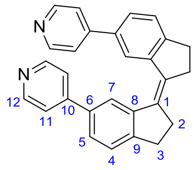

Bromide (*Z*)-**3** (501 mg, 1.28 mmol) and 4-pyridinylboronic acid (495 mg, 4.02 mmol) dissolved in in 9:1 toluene:EtOH (25 mL) and 2M aq. Na_2CO_3 (5 mL) was added. The solution was degassed by bubbling a stream of nitrogen for 30 min. $Pd(PPh_3)_4$ (147 mg, 0.127 mmol) was added in one portion and the mixture degassed for a further 10 min. The reaction was heated to 100 °C and stirred for 16 h. The mixture was then cooled to rt, diluted with CH_2Cl_2 (150 mL) and washed with H_2O (100 mL). The aqueous was back extracted with CH_2Cl_2 (50 mL) and the combined organic extractions dried ($MgSO_4$) and filtered. The filtrate was concentrated *in vacuo* and the residue purified by flash silica chromatography, eluting with 0

– 5% MeOH in CH_2Cl_2. The resultant solid was triturated in Et_2O (30 mL), filtered and air-dried to afford the title compound as an off-white powder (257 mg, 52%).

^{1}H NMR (500 MHz, $CDCl_3$) δ 8.45 (4H, dd, *J* = 4.5, 1.8 Hz, 12-C*H*), 8.39 (2H, s, 7-C*H*), 7.44 (2H, d, *J* = 7.8 Hz, 5-C*H*), 7.41 (2H, d, *J* = 7.8 Hz, 4-C*H*), 7.29 (4H, dd, 4.5, 1.8 Hz, 11-C*H*), 3.09 – 3.05 (4H, m, 3-CH_2), 2.92 – 2.89 (4H, m, 2-CH_2).

^{13}C NMR (126 MHz, $CDCl_3$) δ 150.3 (12-*C*H), 149.6 (9-*C*), 148.7 (10-*C*), 141.5 (8-*C*), 136.0 (6-*C*), 135.4 (1-*C*), 126.4 (5-*C*H), 126.2 (4-*C*H), 122.1 (7-*C*H), 121.6 (11-*C*H), 34.9 (2-*C*H), 30.6 (3-*C*H).

ν_{max} / cm^{-1} (compressed solid) 3066 (w), 3027 (w), 2939 (w), 2905 (w), 2840 (w), 1596 (s), 1543 (m), 1472 (m), 1446 (w), 1422 (w), 1406 (w), 1268 (w), 1220 (w), 992 (w), 809 (s), 730 (m), 613 (w), 606 (w), 533 (w).

ESI-HRMS for $C_{28}H_{23}N_2^+$ $[M+H]^+$ calculated: 387.1856, found 387.1841.

(*Z*)-4,4'-(2,2',3,3'-tetrahydro-[1,1'-biindenylidene]-6,6'-diyl)bis(1-methylpyridin-1-ium) iodide, (*Z*)-1

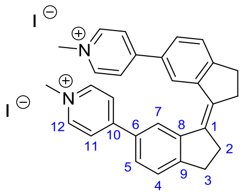

Pyridine (*Z*)-**4** (20 mg, 0.52 mmol), was dissolved in anhydrous DMF (2 mL) and methyl iodide (60 μL, 0.96 mmol) was added. The reaction was heated to 90 °C and stirred for 1 h. The mixture was then cooled to rt and concentrated *in vacuo.* The residue was suspended acetone (5 mL), filtered and the dark solid air dried to afford the title compound (22 mg, 65%). **^{1}H NMR** (500 MHz, DMSO-d_6) δ 8.82 (4H, d, *J* = 6.5 Hz, 12-C*H*), 8.64 (2H, d, *J* = 1.9 Hz, 7-C*H*), 8.31 (4H, d, *J* = 6.5 Hz, 11-C*H*), 7.97 (2H, dd, *J* = 7.9, 1.9 Hz, 5-C*H*), 7.66 (2H, d, *J* = 7.9 Hz, 4-C*H*), 4.28 (6H, s, 13-CH_3), 3.13 – 3.08 (4H, m, 3-CH_2), 2.95 – 2.92 (4H, m, 2-CH_2).

^{13}C NMR (126 MHz, DMSO-d_6) δ 154.3 (10-*C*), 153.0 (9-*C*), 145.4 (12-*C*H), 141.1 (8-*C*), 135.5 (1-*C*), 131.4 (6-*C*), 127.8 (5-*C*H), 126.9 (4-*C*H), 123.5 (11-*C*H), 121.9 (7-*C*H), 47.0 (13-*C*H_3), 34.5 (2-*C*H_2), 30.1 (3-*C*H_2).

ν_{max} / cm^{-1} (compressed solid) 3015 (w), 2929 (w), 1639 (s), 1594 (m), 1558 (m), 1522 (m), 1473 (m), 1443 (m), 1425 (m), 1321 (m), 1279 (m), 1203 (m), 1194 (m), 819 (S), 722 (w), 599 (m), 508 (s), 425 (s).

ESI-HRMS $C_{30}H_{28}N_2^{2+}$ $[M]^{2+}$ calcd: 208.1121, found 208.1125.

6-(pyridin-4-yl)-2,3-dihydro-1H-inden-1-one, 7

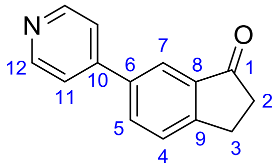

6-bromo-1-indanone (249 mg, 1.18 mmol) and 4-pyridinylboronic acid (256 mg, 2.08 mmol) were dissolved in toluene (45 mL) and EtOH (5 mL) and 2M aqueous Na_2CO_3 (10 mL) was added. The solution was degassed by bubbling a stream of nitrogen for 30 min. $Pd(PPh_3)_4$ (137 mg, 0.119 mmol) was added in one portion and the mixture degassed for a further 10 min. The reaction was heated to 100 °C and stirred for 17 h. The mixture was then cooled to rt, diluted with CH_2Cl_2 (50 mL) and washed with water (50 mL). The aqueous was back extracted with CH_2Cl_2 (2 x 50 mL) and the combined organic extractions dried ($MgSO_4$) and filtered. The filtrate was concentrated *in vacuo* and the residue purified by flash silica chromatography, eluting with 0 – 5% MeOH in CH_2Cl_2, to afford a white solid. This was suspended in Et_2O (20 mL), stirred vigorously for 10 min, and the remaining insoluble solid filtered and air-dried to afford ketone **7** as an off-white powder (70 mg, 28%).

^{1}H NMR (500 MHz, DMSO-d_6) δ 8.73 – 8.61 (2H, dd, J = 4.6, 1.6 Hz, 12-C*H*), 8.12 (2H, dd, J = 8.0, 1.9 Hz, 5-C*H*), 8.01 (2H, d, J = 1.9 Hz, 7-C*H*), 7.79 (2H, dd, J = 4.6, 1.6 Hz, 11-C*H*), 7.75 (2H, d, J = 8.0 Hz, 4-C*H*), 3.29 – 3.07 (2H, m, 3-CH_2), 2.77 – 2.59 (2H, m, 2-CH_2). **^{13}C NMR** (126 MHz, DMSO-d_6) δ 206.0 (1-*C*O), 156.2 (9-*C*), 150.3 (12-*C*H), 146.1 (10-*C*), 137.6 (8-*C*), 136.5 (6-*C*), 133.2 (5-C*H*), 127.9 (4-*C*H), 121.4 (11-*C*H), 120.9 (7-*C*H), 36.3 (2-*C*H_2), 25.4 (3-*C*H_2). **ν_{max} / cm^{-1}** (compressed solid) 3077 (w), 3058 (w), 3032 (w), 2924 (w), 2915 (w), 1700 (s), 1615 (m), 1594 (s), 1481 (m), 1437 (s), 1402 (m), 1302 (m), 1237 (m), 1178 (m), 1028 (m), 991 (w), 818 (s), 803 (s), 647 (m), 560 (s), 468 (s). **ESI-HRMS** $C_{14}H_{12}NO^+$ $[M+H]^+$ calcd: 210.0913, found 210.0914.

6 NMR spectra of novel compounds

Compound (*E*)-4: ^{1}H NMR (500 MHz, $CDCl_3$)

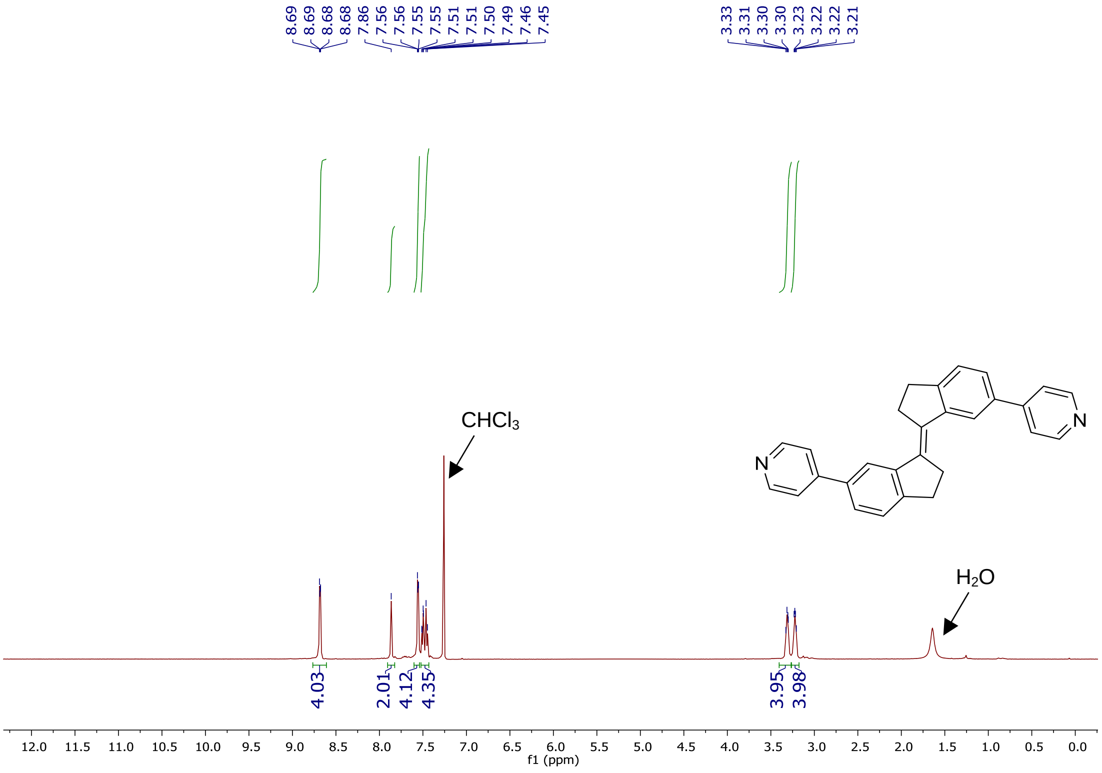

Compound (*E*)-4: ^{13}C NMR (126 MHz, $CDCl_3$)

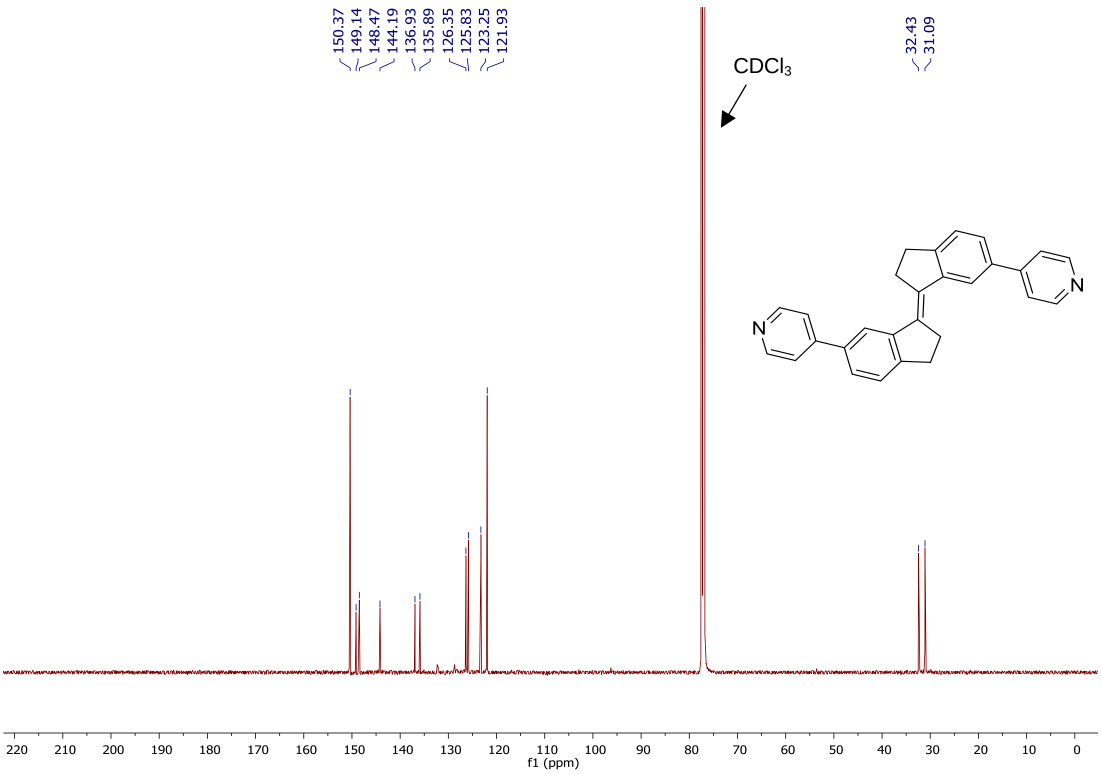

Compound (*Z*)-4: ^{1}H NMR (500 MHz, $CDCl_3$)

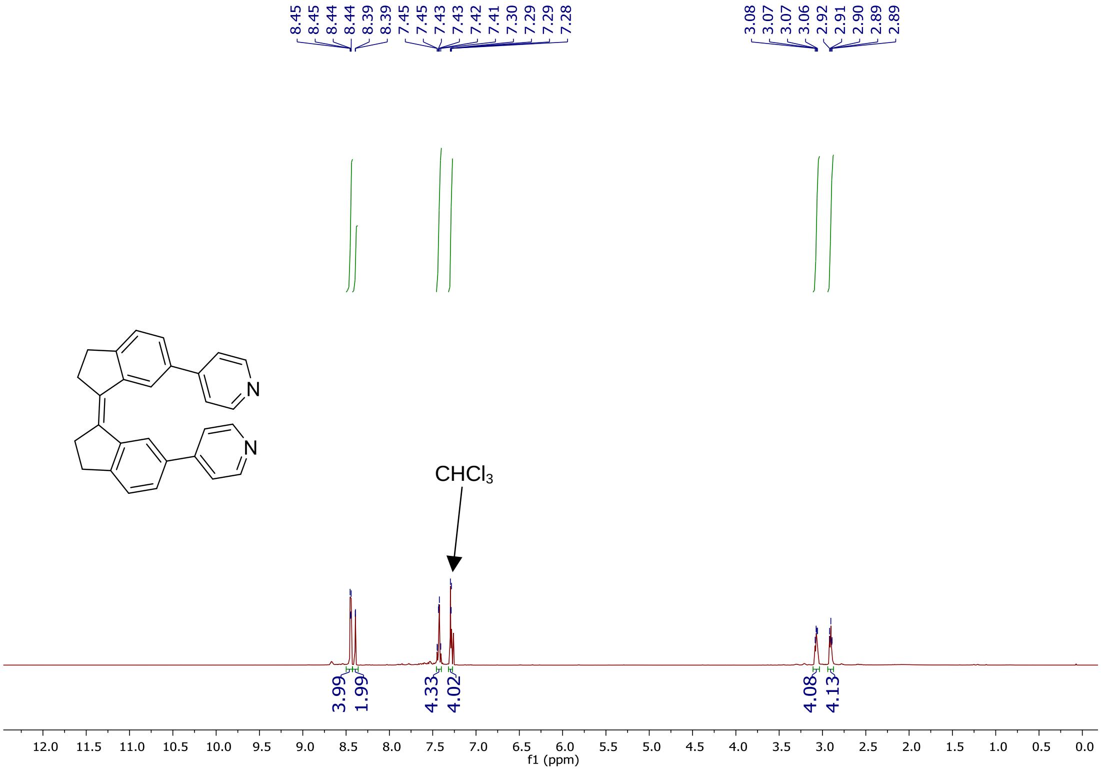

Compound (*Z*)-4: ^{13}C NMR (126 MHz, $CDCl_3$)

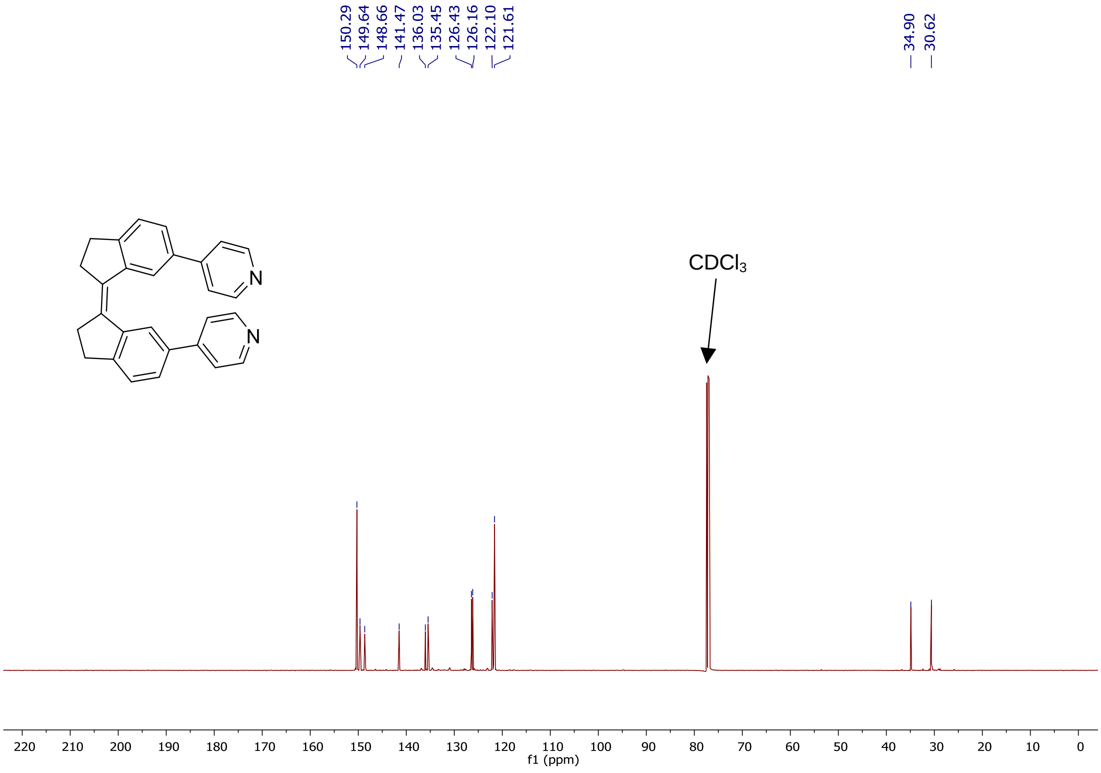

Compound (*E*)-1: ^{1}H NMR (500 MHz, DMSO-d_6)

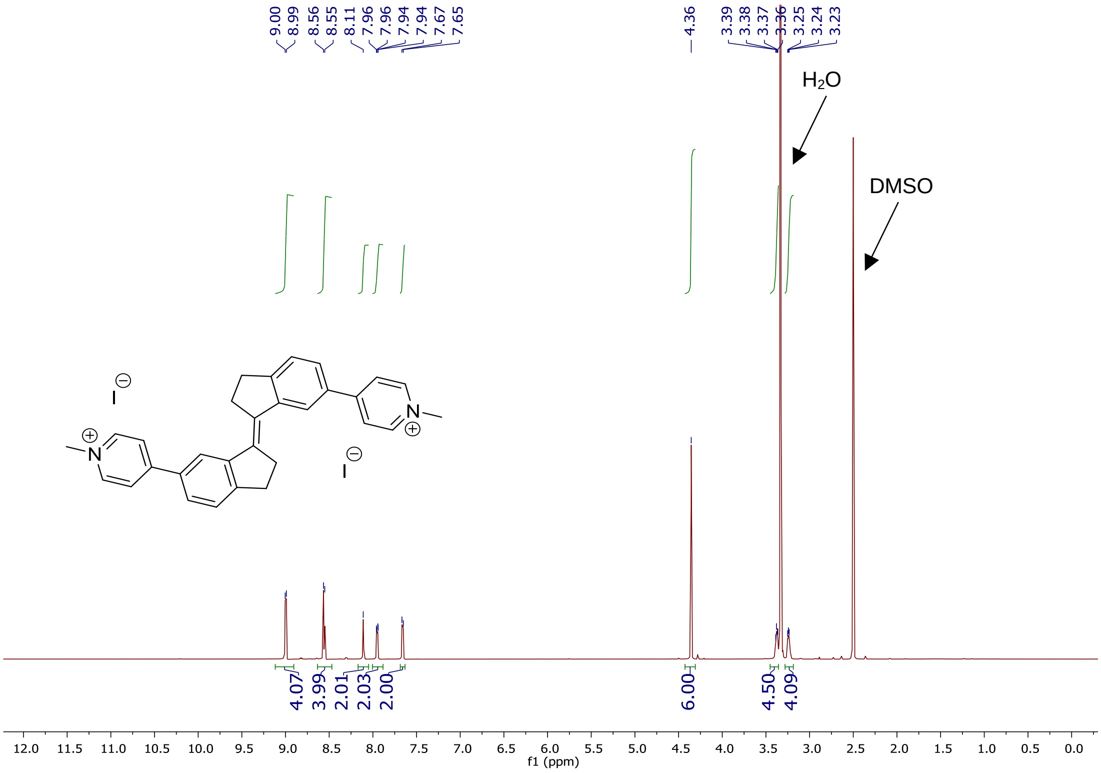

Compound (*E*)-1: ^{13}C NMR (126 MHz, DMSO-d_6)

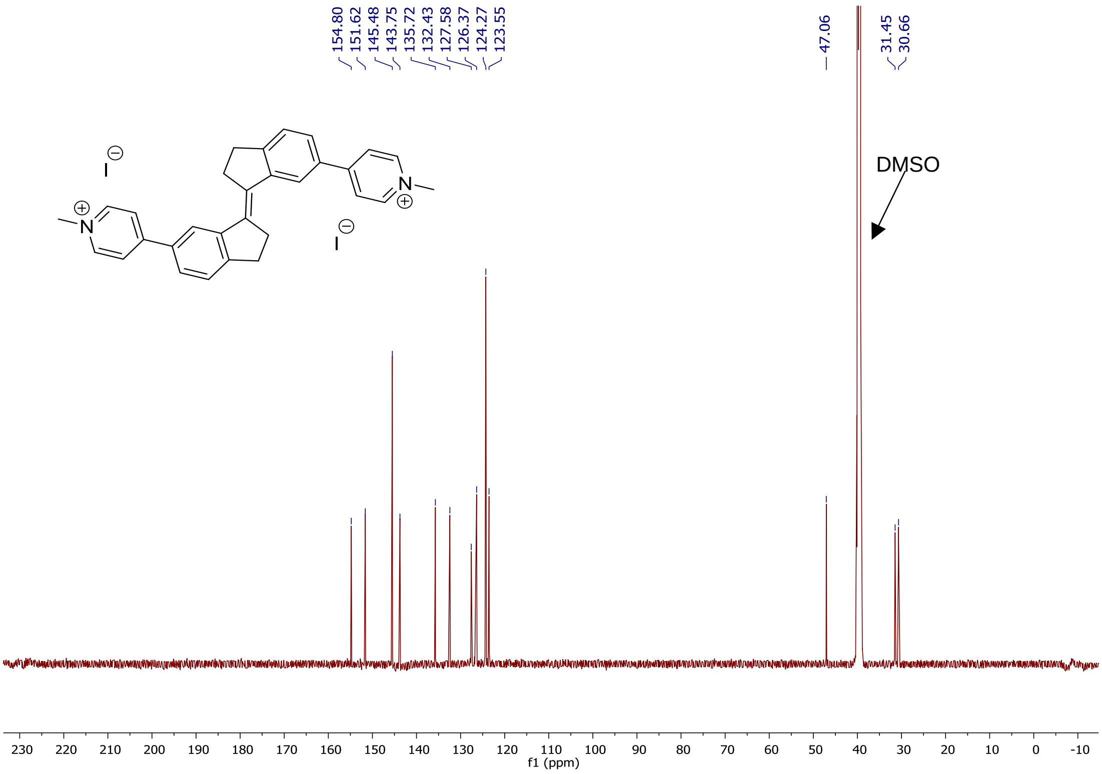

Compound (*Z*)-1: ^{1}H NMR (500 MHz, DMSO-d_6)

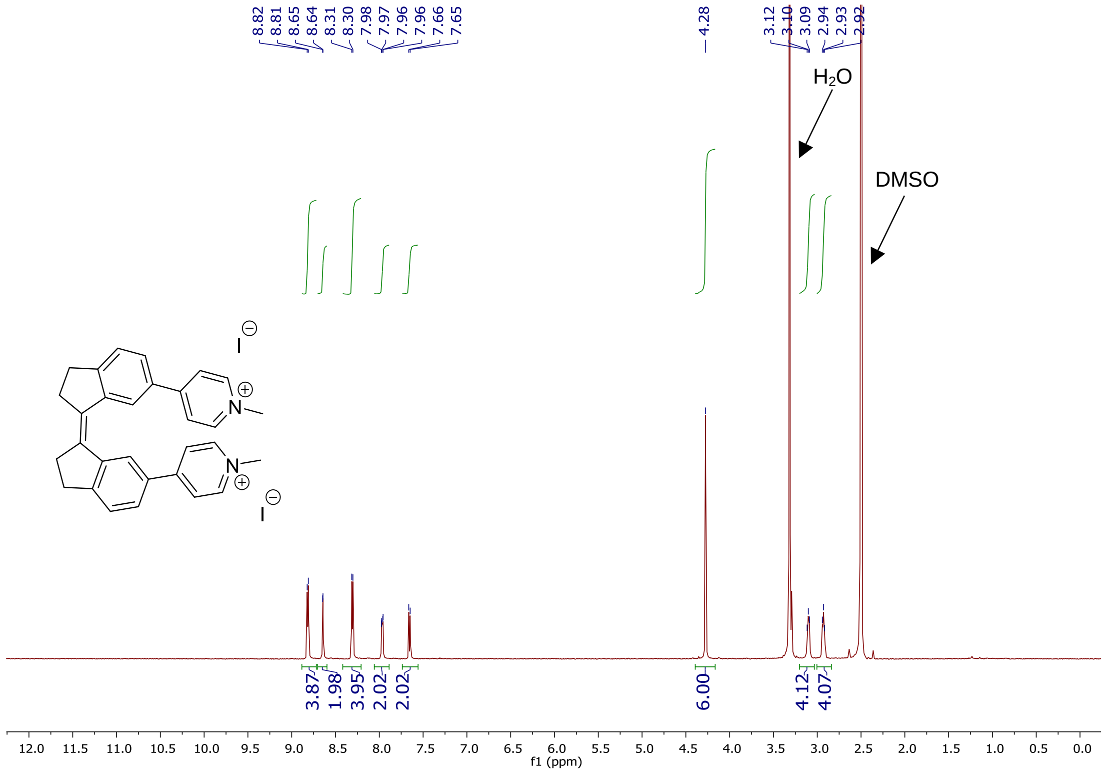

Compound (*Z*)-1: ^{13}C NMR (126 MHz, DMSO-d_6)

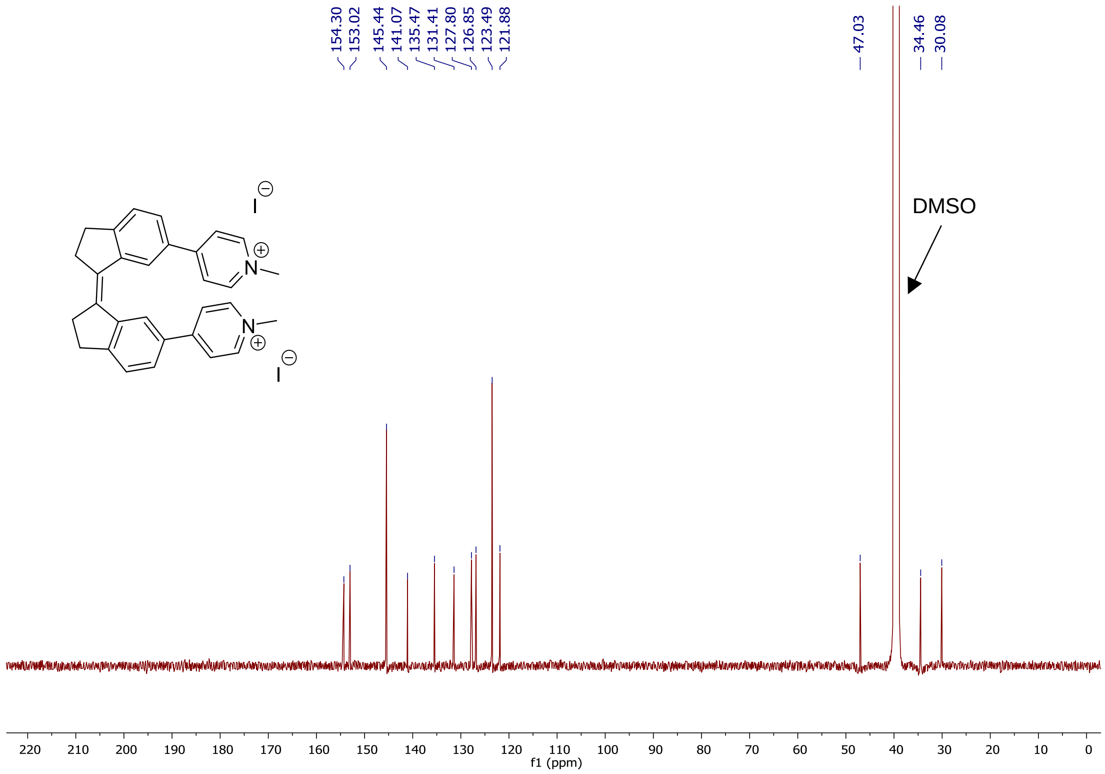

Compound 7: ^{1}H NMR (500 MHz, DMSO-d_6)

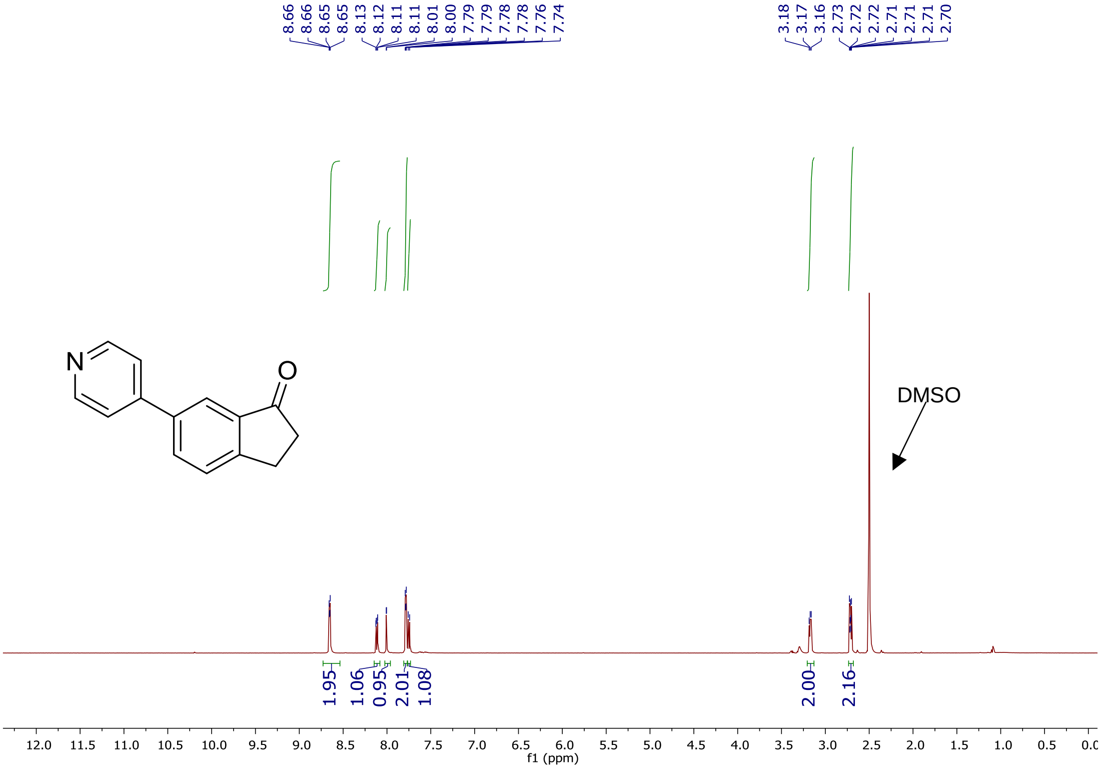

Compound 7: ^{13}C NMR (126 MHz, DMSO-d_6)

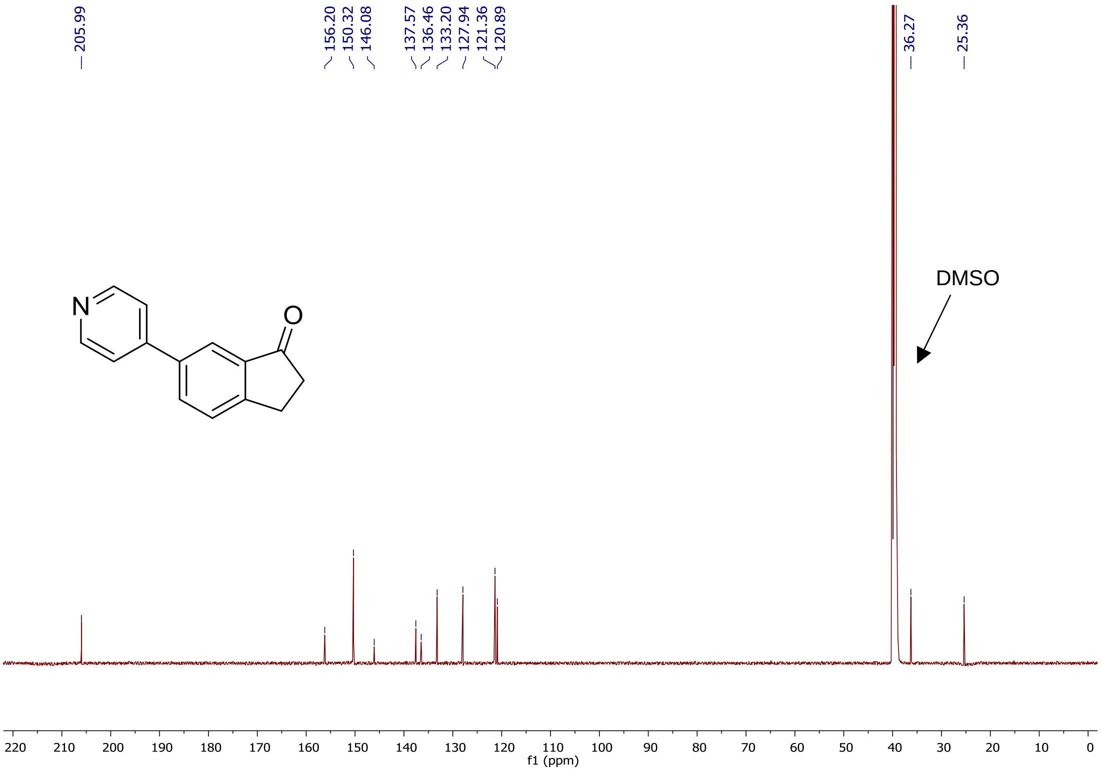

7 References

[1] A. De Cian, L. Guittat, M. Kaiser, B. Saccà, S. Amrane, A. Bourdoncle, P. Alberti, M. P. Teulade-Fichou, L. Lacroix, J. L. Mergny, *Methods* **2007**, *42*, 183–195.

[2] M. D. Pelta, G. A. Morris, M. J. Stchedroff, S. J. Hammond, *Magn. Reson. Chem.* **2002**, *40*, S147-S152.

[3] Y. Wang, D. J. Patel, *Structure* **1993**, *1*, 263–282.

[4] O. Trott, A. J. Olson, *J. Comput. Chem.* **2010**, *31*, 455–461.

[5] B. Hess, C. Kutzner, D. Van Der Spoel, E. Lindahl, *J. Chem. Theory Comput.* **2008**, *4*, 435–447.

[6] J. Wang, R. M. Wolf, J. W. Caldwell, P. A. Kollman, D. A. Case, *J. Comput. Chem.* **2004**, *25*, 1157–1174.

[7] J. Wang, W. Wang, P. A. Kollman, D. A. Case, *J. Mol. Graph. Model.* **2006**, *25*, 247–260.

[8] C. I. Bayly, P. Cieplak, W. D. Cornell, P. A. Kollman, *J. Phys. Chem.* **1993**, *97*, 10269–10280.

[9] W. L. Jorgensen, J. Chandrasekhar, J. D. Madura, R. W. Impey, M. L. Klein, *J. Chem. Phys.* **1983**, *79*, 926–935.

[10] J. Åqvist, *J. Phys. Chem.* **1990**, *94*, 8021–8024.

[11] G. Bussi, D. Donadio, M. Parrinello, *J. Chem. Phys.* **2007**, *126*, 14101.

[12] G. Bussi, T. Zykova-Timan, M. Parrinello, *J. Chem. Phys.* **2009**, *130*, 74101.

[13] H. J. C. Berendsen, J. P. M. Postma, W. F. Van Gunsteren, A. Dinola, J. R. Haak, H. J. C. Berendsen, J. P. M. Postma, W. F. Van Gunsteren, A. Dinola, J. R. Haak, *J. Chem. Phys.* **2012**, *3684*, 926–935.

[14] S. Nosé, *Mol. Phys.* **2002**, *100*, 191–198.

[15] W. G. Hoover, *Phys. Rev. A* **1985**, *31*, 1695–1697.

[16] M. Parrinello, A. Rahman, *J. Appl. Phys.* **1981**, *52*, 7182–7190.

[17] T. Darden, D. York, L. Pedersen, T. Darden, D. York, L. Pedersen, **1998**, *10089*, 1–5.

[18] A. Barducci, G. Bussi, M. Parrinello, *Phys. Rev. Lett.* **2008**, *100*, 1–4.

[19] G. A. Tribello, M. Bonomi, D. Branduardi, C. Camilloni, G. Bussi, *Comput. Phys. Commun.* **2014**, *185*, 604–613.

[20] A. B. Pangborn, M. A. Giardello, R. H. Grubbs, R. K. Rosen, F. J. Timmers, *Organometallics* **1996**, *15*, 1518–1520.

[21] W. C. Still, M. Kahn, A. Mitra, *J. Org. Chem.* **1978**, *43*, 2923–2925.

[22] S. J. Wezenberg, B. L. Feringa, *Org. Lett.* **2017**, *19*, 324–327.
